# Positional identification of a candidate gene for *MALE STERILITY 2* (*MS2*) by linkage mapping and transcriptomic data in *Cryptomeria japonica* D. Don

**DOI:** 10.1186/s12864-026-12907-4

**Published:** 2026-05-19

**Authors:** Saneyoshi Ueno, Yoichi Hasegawa, Tokuko Ujino-Ihara, Momi Tsuruta, Hiroyuki Kakui, Junji Iwai, Satoko Hirayama, Katsushi Yamaguchi, Shuji Shigenobu, Takeshi Fujino, Yutaka Suzuki, Masahiro Kasahara, Yoshinari Moriguchi

**Affiliations:** 1 Department of Forest Molecular Genetics and Biotechnology, Forestry and Forest Products Research Institute, Forest Research and Management Organization, 1 Matsunosato, Tsukuba, Ibaraki 305-8687 Japan; 2 Institute for Sustainable Agro-ecosystem Services, Graduate School of Agricultural and Life Sciences, The University of Tokyo, 1-1-1 Midoricho, Nishitokyo, Tokyo 188-0002 Japan; 3 Niigata Prefectural Forest Research Institute, 2249-5 Unotoro, Murakami, Niigata 958-0264 Japan; 4 Trans-Scale Biology Center, National Institute for Basic Biology, Okazaki, Aichi 444-8585 Japan; 5 Graduate School of Frontier Sciences, The University of Tokyo, 5-1-5 Kashiwanoha, Kashiwa, Chiba 277-8561 Japan; 6 Graduate School of Science and Technology, Niigata University, 8050 Ikarashi 2-no-cho, Nishi-ku, Niigata, 950-2181 Japan; 7 Current address: Department of Agriculture, Faculty of Agriculture, Niigata University, 8050 Ikarashi 2-no-cho, Nishi-ku, Niigata, 950-2181 Japan; 8 Current address: Graduate School of Environmental, Life, Natural Science and Technology, Okayama University, Okayama, Japan; 9 Current address: Forestry Conservation Division, Department of Agriculture, Forestry and Fisheries, Niigata Prefectural Government, Niigata, Japan; 10 Current address: Forestry Administration Division, Department of Agriculture, Forestry and Fisheries, Niigata Prefectural Government, Niigata, Japan

**Keywords:** *Cryptomeria japonica*, Male sterility, GELP, GDSL esterase/lipase, Map-based cloning, Pollen development, Conifer genomics, Pollinosis

## Abstract

**Background:**

Japanese cedar (*Cryptomeria japonica* D. Don) is a major plantation species in Japan, but its abundant pollen production is a primary cause of seasonal allergic rhinitis (pollinosis). To mitigate this public health issue, the use of male-sterile cultivars has been promoted. Five types of recessive male-sterile mutations (*ms1*–*ms5*) have been identified, and the causal genes and mutations for *MS1* and *MS4* have been elucidated. However, the gene responsible for MS2-type male sterility remains unknown.

**Results:**

We aimed to identify the candidate gene responsible for MS2-type male sterility using a map-based cloning strategy. High-resolution linkage mapping localized the *MS2* locus to a 1.56 cM interval on linkage group 5, corresponding to an 8.64 Mb region of the reference genome. Ninety-one genes in this region were subjected to functional annotation, gene expression analysis, and mutation screening. Among these, a single gene, SUGI_0493010, encoding a GDSL-type esterase/lipase protein (GELP), fulfilled all three criteria: it showed homology to pollen development genes in *Arabidopsis thaliana*, was specifically expressed in male strobili, and carried a deleterious amino acid substitution (S40F) within the predicted catalytic domain in *ms2* mutant. The same mutation was also detected in a heterozygous individual (*Ms2/ms2*) from a separate breeding population, whose genotype was confirmed through progeny testing. Structural annotation revealed that the affected serine residue lies within the conserved GDSL motif, suggesting a functional disruption of enzymatic activity.

**Conclusions:**

Our results strongly suggest that SUGI_0493010 (*GELP*) is the candidate gene for MS2-type male sterility in *C. japonica*. This finding enhances our understanding of male sterility mechanisms in conifers and provides a valuable genetic resource for breeding pollen-free trees. The study also demonstrates the effectiveness of combining genetic mapping with transcriptomic and mutational data in forest tree genomics.

**Supplementary Information:**

The online version contains supplementary material available at 10.1186/s12864-026-12907-4.

## Introduction

 Japanese cedar (*Cryptomeria japonica* D. Don), or sugi, is one of the most widely planted tree species in Japan due to its economic importance in the forest industry, occupying about 40% of artificial forests in Japan [1]. The timber has been used not only in house construction, but also in traditional crafts and daily-use wooden products, including furniture, chopsticks, sake barrels, and shrine architecture. However, large-scale sugi plantations have contributed to a major public health issue: pollen allergy or sugi pollinosis. This seasonal allergy has become increasingly prevalent, with nationwide surveys indicating that nearly 40% of the population was affected in 2019 [2]. The primary source of the allergen is the massive production of pollen from male strobili during the spring season, varying widely among studies, ranging from approximately 100,000 to 656,000 grains per single male strobilus depending on the clone and environmental conditions [3–6]. In order to reduce pollen emissions at the source, increasing attention has been directed toward the utilization of male-sterile cultivars in afforestation.

Genetic characterization of male-sterile mutants in *C. japonica* has provided a solid foundation for this approach. Recessive mutations causing male sterility have been classified into five types (*MS1*–*MS5*) based on the specific developmental stage at which pollen formation is disrupted. These classifications were established through detailed cytological observations [7–10] and linkage analyses using controlled crosses [11–13]. Among these, the MS1-type has been the most extensively studied [8, 14, 15] and has already been utilized in public afforestation initiatives [16]. Pollen-free sugi trees homozygous for the *ms1* allele (*ms1/ms1*) have been propagated and planted to reduce airborne pollen emission from sugi forests. Recent molecular studies [17, 18] have further advanced our understanding of the genetic basis of male sterility in *C. japonica*, particularly for *MS1* and *MS4*. *MS1*, which is associated with complete pollen abortion at the tetrad stage, has been linked to a gene involved in lipid transportation. Similarly, *ms4*, which disrupts pollen formation during the microspore stage, has been associated with a gene orthologous to *Arabidopsis thaliana TKPR1*, a key enzyme in sporopollenin biosynthesis [19]. These findings have provided valuable insights into the molecular mechanisms underlying pollen development and laid the foundation for marker-assisted selection of male-sterile trees in breeding programs [20, 21].

**Fig. 1:**
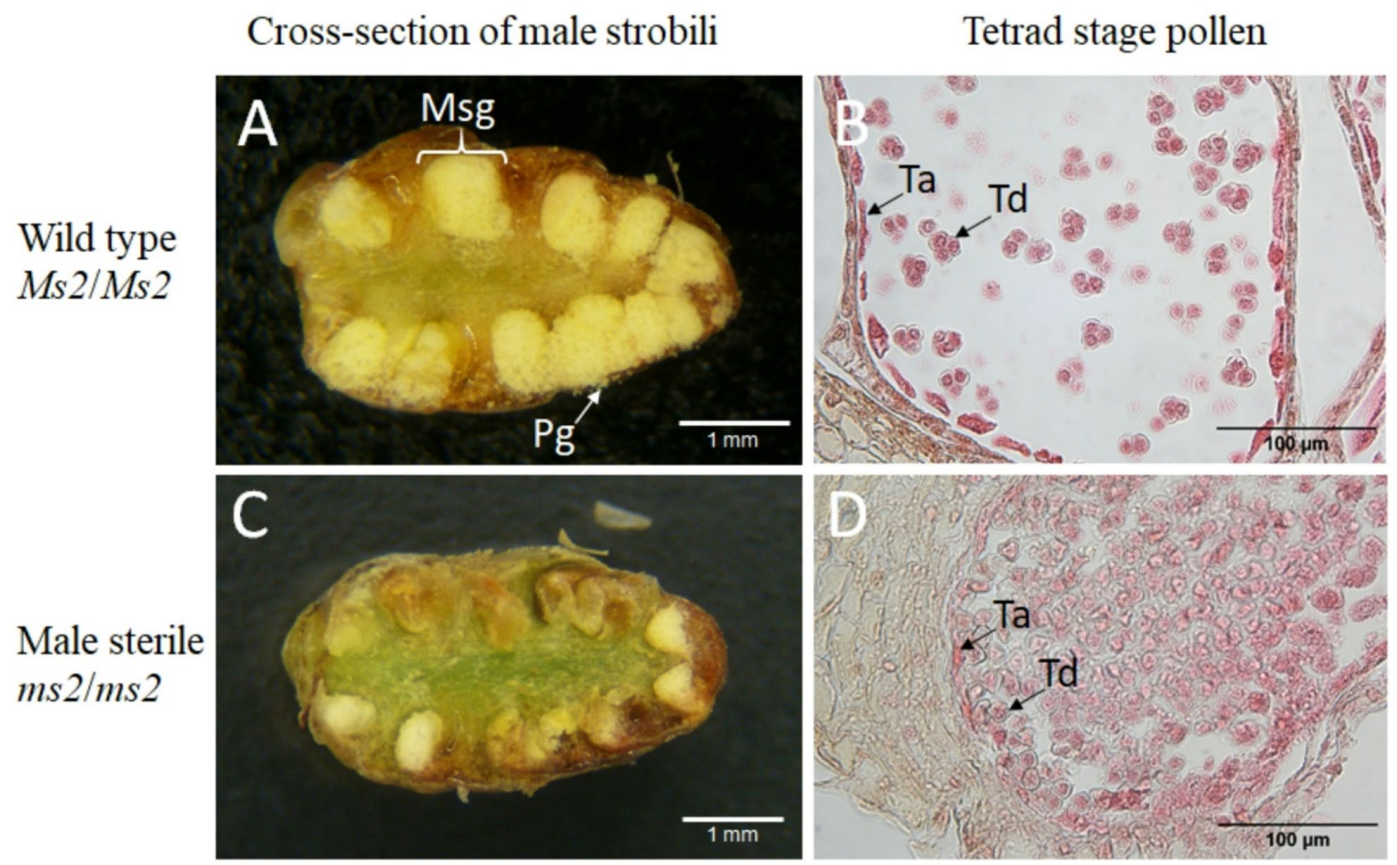
Comparison of male strobili and tetrad stage pollen between fertile and MS2-type male-sterile individuals of *Cryptomeria japonica*. Representative images showing the morphological and cytological differences between wild-type (*Ms2*/*Ms2*) and MS2-type male-sterile (*ms2*/*ms2*) individuals. Left: Cross-sections of male strobili. Wild-type (**A**) microsporangia (Msg) were packed with discrete pollen grains (Pg), while those of the *ms2* mutant (**C**) lacked normal grains and contained an amorphous, paste-like mass of microspores instead. Right: Light microscopic image of pollen at the tetrad stage. In fertile individuals, pollen development proceeds normally (**B**), while in sterile individuals (`Shindai-1`), tetrads exhibit developmental abnormality with clumping and dense aggregation (**D**). Scale bars for the cross-sections are estimated based on the approximate size of the male strobilus (~4 mm) and are intended for reference only. Msg, microsporangium; Pg, pollen grains; Ta, tapetum; Td, tetrad

Despite these advances, the molecular evidence remains uneven across MS types, and MS2-type male sterility remains unresolved. *MS2* is characterized by developmental abnormality from the tetrad stage, as previously reported in [10] (Fig. 1) and was first identified in a naturally occurring mutant known as `Shindai-1,` selected from an artificial forest in Niigata Prefecture in 1998 [9]. Previous studies have localized the *MS2* locus to linkage group 5 (LG5) [11, 22], but the causal gene has not yet been identified. Understanding the genetic mechanism underlying *MS2* is essential not only for expanding the tools for marker-assisted breeding but also for deepening our knowledge of reproductive development in conifers.

In this study, we aimed to identify the candidate gene responsible for MS2-type male sterility using a map-based cloning approach. By integrating high-resolution linkage mapping, RNA-Seq-based expression profiling, and mutation screening with functional prediction, we successfully narrowed down the candidate region and identified a GDSL-type esterase/lipase gene (*GELP*) as the most likely candidate gene. Our findings provide important insights into the molecular basis of male sterility in *C. japonica* and offer a genetic resource for the practical development of pollen-free sugi trees.

## Materials and methods

### Plant materials

The MS2-type male-sterile individual `Shindai-1` was originally selected from an artificial forest in Niigata Prefecture, Japan [9]. This individual was used as the seed parent to produce a mapping family (S1-2), which was generated by crossing `Shindai-1` (*ms2*/*ms2*) and `S1NK4` (*Ms2*/*ms2*) as female and male parent, respectively [22]. The `S1NK4` is an offspring (F1) resulting from a cross between `Shindai-1` and `Nakakubiki-4.` Among the offspring of this family, 129 individuals were used for refinement of the linkage map in our previous study [22]. For further linkage-based refinement of the *MS2* locus, we selected 95 of these 129 individuals (31 male-sterile and 64 male-fertile). Because the *MS2* male-sterile phenotype can be difficult to distinguish in some individuals, only those with clearly identifiable phenotypes were included in the analysis. Phenotypes were determined based on multiple (at least two times) field observations of male strobili and microscopic examination of dissected male strobili. These observations were carried out from January to March in 2010, 2011, and 2012. In addition, another individual from Niigata Prefecture, `Gosenshi-1,` was included only to evaluate the consistency of the candidate mutation identified in this study. This individual had been previously identified as *Ms2*/*ms2* through controlled crossing experiments with `Shindai-1` (*ms2*/*ms2*), which yielded an approximately 1:1 segregation of male-fertile and male-sterile progeny (49 fertile vs. 54 sterile seedlings) [23],

### RNA-Seq data collection and analysis

Male strobili were collected from fertile and sterile individuals of the S1-2 family growing at Chiyoda nursery, FFPRI (36.184269° N, 140.217564° E) at two time points (October 17th and November 6th, 2019) for RNA-Seq analysis (Additional file 1). These dates were selected to span the developmental window in which the *MS2* phenotype is first observed, i.e., around the tetrad-to-early microspore transition. For each time point, male strobili were sampled from both fertile (wild-type) and MS2-sterile individuals, and one RNA-Seq library was prepared per sample, resulting in a total of 15 libraries (October: five sterile and three fertile; November: four sterile and three fertile; Additional file 1). Individuals were chosen based on field accessibility of trees primarily located along the stand edge.

The male strobilus samples were immediately frozen in liquid N_2_ at the field, and stored at −80 °C until RNA extraction. The total RNA was extracted from the male strobili using the modified cetyltrimethylammonium bromide (CTAB) method [23]. RNA-Seq was performed following Macrogen’s regular workflow (Macrogen Japan Corp., Kyoto, Japan). Briefly, high-throughput sequencing libraries were constructed using the TruSeq stranded mRNA Library Kit (Illumina, San Diego, CA, USA) following the quality control of RNA. Each library was sequenced by the Illumina platform (NovaSeq6000) at 100 bp paired-end reads (on average 7.3 Gbp per sample). Raw reads were processed using fastp [24] to remove adapter sequences and low-quality regions. Cleaned reads were aligned using bwa mem (ver. 0.7.17-r1188) with default parameters. For the analyses reported in this study, all RNA-Seq libraries were aligned to the *Cryptomeria japonica* SUGI_1 *permissive gene set* [25] as the common mapping reference. The earlier transcript reference CJ3006NRE [27] had been used in marker development and preliminary analyses before the chromosome-scale SUGI_1 reference became available, but those preliminary analyses were not combined with the downstream analyses presented here. Instead, BAM files aligned to the SUGI_1 *permissive gene set* were subsequently utilized as a common resource for downstream analyses—including SNP identification, gene expression analysis (including DEG analysis), and detection of deleterious mutations—all of which focused on the SUGI_1 *standard gene set* [25] consisting of 55,246 annotated genes. Gene-level read counts were obtained using featureCounts [26], and differential expression statistics (baseMean, log2 fold change, and adjusted P-values) between fertile and sterile male strobili were calculated separately for each sampling date using DESeq2 [27] with default settings. In this study, the RNA-Seq data set was used primarily as a discovery resource to identify genes expressed in male strobili, characterize their developmental expression patterns, and screen coding variants within the *MS2* interval for marker development, rather than for precise quantitative estimation of fold changes.

### Marker development using RNA-Seq data

Based on our previous linkage map [22], the *MS2* locus was initially localized to three genomic contigs—ctg668, ctg930, and ctg820—in the *C. japonica* draft genome ver. 0.1 (Fujino et al., unpublished) (Additional file 2) by performing BLASTN searches using Axiom SNPType assay probe sequences (GEO accession GPL25617). At that time, a chromosome-scale genome assembly and gene annotations were not yet available. Therefore, we identified genes on these contigs by performing BLASTN searches using CJ3006NRE reference transcript sequences from our previous study [28] as queries. To improve the accuracy of gene models and prioritize polymorphisms, spliced alignments were performed using Splign [29] to infer exon–intron structures. After the chromosome-scale assembly SUGI_1 became available [26], we additionally aligned these draft contigs to SUGI_1 to confirm their chromosomal locations.

Polymorphisms between fertile and sterile individuals were identified using RNA-Seq data. BAM files were grouped by phenotype (fertile or sterile), and SNPs were called using VarScan (v2.4.3) [30, 31] (mpileup2snp; --min-coverage 20 --min-reads2 5 --min-avg-qual 30), generating VCF files for downstream analysis. Within each target gene, consensus sequences incorporating heterozygous sites were created using ‘bcftools consensus’ (v1.9) [32] with IUPAC ambiguity codes. Based on the exon–intron structures inferred by Splign, intronic regions were replaced with Ns to generate template sequences for SNP assay design, and these sequences were then post-processed to fit the input format of Fluidigm SNPType Assay software D3 (Standard Biotools).

### SNP genotyping and linkage map refinement

Candidate SNPs were evaluated using D3 to determine whether functional primer pairs could be designed. Only SNPs that passed the D3 design criteria were selected for genotyping. Genotyping was carried out using the Fluidigm EP1 system with a 48.48 Dynamic Array according to the manufacturer’s instructions. Genotypes were called with Fluidigm SNP Genotyping Analysis software (v4.5.1). The resulting genotype data were used to construct a refined linkage map. Linkage analysis was conducted using the maximum likelihood mapping algorithm in JoinMap ver. 5 (Kyazma, Wageningen, The Netherlands), assuming a backcross (BC)-type population (nn × np), as previously described [22]. Once a chromosome-scale genome assembly (SUGI_1) became available [25], the newly developed markers were mapped onto chromosome 5 (chr5).

### Candidate gene identification by functional annotation

To identify candidate genes within the *MS2* region described above, nucleotide sequences corresponding to the *standard gene set* [25] in this region (Additional file 3) were subjected to BLASTX searches (E-value cutoff: 1e-5) against the TAIR10 protein database of *Arabidopsis thaliana* [33]. Genes in *C. japonica* showing homology to 372 *Arabidopsis* gene loci annotated with GO:0009555 (pollen development) were selected as functional (homology-based) candidates.

### Gene expression analysis for candidate gene prioritization

Gene expression analysis was performed to identify candidate genes expressed in fertile individuals within the 8.64 Mb interval on chromosome 5. Gene expression levels were quantified using transcripts per million (TPM) values calculated from the RNA-Seq data using featureCount [26], based on BAM files aligned to the SUGI_1 *permissive gene set*. TPM values were calculated across all annotated transcripts, and only genes corresponding to the SUGI_1 *standard gene set* (55,246 genes) were retained for downstream analysis. Genes with TPM values > 1 in at least one fertile individual were considered expressed and retained as *MS2* candidate genes.

For differential gene expression analysis, RNA-Seq libraries from eight individuals (five male-sterile and three male-fertile) were used. Because seven of these individuals were sampled at both the October and November time points, the DEG dataset comprised 15 RNA-Seq libraries. One male-sterile individual (`Y621`) was sampled only in October (Additional file 1). Differential gene expression analysis was conducted separately for each sampling time point using DESeq2 [27] with raw count data from the SUGI_1 *permissive gene set*: the October dataset included eight libraries (five male-sterile and three male-fertile), whereas the November dataset included seven libraries (four male-sterile and three male-fertile). For interpretation, we focused on the MS2-neighboring genes. Genes with an adjusted *p*-value < 0.01 were considered significantly differentially expressed.

### Identification of deleterious mutations and validation in additional material

To investigate whether deleterious mutations were responsible for MS2-type male sterility, polymorphisms detected within candidate genes were evaluated using PROVEAN (v1.1.3) [34]. Variant information from the previously generated VCF file was used to create sterile (ALT) haplotype sequences by using ‘bcftools consensus’ (ver. 1.9) [32], translated into protein sequences by transeq in EMBOSS suite [35], and the resulting amino acid substitutions were analyzed for their functional effects using PROVEAN (v1.1.3). Variants with a PROVEAN score below −2.5 were classified as deleterious. Candidate genes with deleterious mutations were prioritized for further investigation.

To assess the structural consequences of the amino-acid substitution identified in the candidate gene by PROVEAN, we generated homology models of the corresponding protein using Phyre2 [36]. The full-length amino-acid sequence of the wild-type was submitted to Phyre2, and the resulting three-dimensional model was used for structural evaluation. Structural effects of the substitution were assessed using Missense3D [37].

To further validate the presence of the identified mutation in an independent genetic background, we also analyzed an additional heterozygous individual, `Gosenshi-1` (*Ms2*/*ms2*), from which male strobili were collected at Niigata Prefectural Forest Research Institute on 22 October 2020 for RNA extraction. RNA extraction and mutation detection for `Gosenshi-1` were performed using the same methods as described above.

### Structural, functional, expressional and phylogenetic profiling of candidate gene

The gene structure of a candidate gene (SUGI_0493010) was determined using the SUGI_1 genome assembly and gene models from the *standard gene set* [25]. To refine transcriptional boundaries, full length cDNA sequence (DDBJ accession number: FX344585) and Iso-Seq reads for male strobili from our previous studies [28, 38] were mapped using minimap2 [39] with options (-ax splice -uf -k14 --secondary = no) and visualized in CLC Genomics Workbench ver. 20 (Qiagen). Transcription start site (TSS) was inferred based on sharp increases in read coverage, and on the prediction by TSSPlant [40] with input of a 3.4-kb upstream sequence from the start codon. The 5′UTR was defined as the region between the TSS and the annotated start codon, while the 3′UTR was defined as the region between the stop codon and the major polyadenylation site inferred from Iso-Seq read ends. The predicted amino acid sequence (364 residues) was annotated using InterPro webtool [41].

In order to get insights into expressional variation among different tissues for the *MS2* candidate gene (SUGI_0493010), CPM (count per million) data were downloaded from the SugiExDB database [42], which contains RNA-Seq expression profiles for various *C. japonica* tissues. Expression levels were visually compared across tissues to explore tissue-preferential expression tendencies.

To assess the temporal expression pattern of the candidate gene, we reanalyzed the microarray dataset [15], which includes male strobilus samples collected at nine defined developmental stages. The candidate gene sequence was BLASTed against the probe sequences, and the probe corresponding to the candidate gene was identified. Normalized probe intensities for the probe corresponding to SUGI_0493010 were then extracted from the published dataset, and temporal changes in its expression pattern were examined.

To further contextualize the *MS2* candidate gene (GDSL esterase/lipase protein: GELP), putative GELP genes were identified from the *C. japonica* genome (*permissive gene set*) using InterProScan v5.55-88.0 [43] to detect conserved protein domains associated with the GDSL esterase/lipase family. Proteins containing GDSL-related InterPro signatures: IPR001087 (Lipase, GDSL), IPR030374 (GDSL-like lipase/acylhydrolase), or Pfam signature: Lipase_GDSL, were retained as candidate GELP genes. Domain descriptions containing GDSL-related keywords (e.g., “GDSL,” “esterase/lipase,” “SGNH hydrolase”) were also used to capture divergent GELP members. To obtain information on secretion signals and membrane topology, SignalP 6.0 [44] and TMHMM 2.0 [45] analyses were conducted for all proteins. SignalP predictions (Sec/SPI signal peptide status and cleavage site) and TMHMM-predicted transmembrane helices were not used as exclusion criteria but were retained as annotation features, because incomplete 5′ gene models often lead to false-negative signal peptide predictions [46].

For phylogenetic analysis of the *C. japonica* GELP family, Pfam domain searches were performed using HMMER (hmmscan) ver. 3.3 [47] against the Pfam-A database (release 34.0) [48], and proteins with hits to Lipase_GDSL (PF00657.24; tlen = 224) were identified. When multiple PF00657 domain hits were detected in a protein, the best hit (lowest i-Evalue) was selected. To reduce the impact of truncated domain fragments on multiple sequence alignment, only PF00657 domain hits covering ≥ 50% of the HMM model, calculated as hmmcov = (hmmto – hmmfrom + 1)/tlen, were retained. The resulting PF00657 domain sequences were aligned using MAFFT v7 [49] with the L-INS-i strategy (mafft --localpair --maxiterate 1000). A neighbor-joining (NJ) tree was inferred from the alignment using MEGA ver. 11 [50].

Finally, we conducted phylogenetic analyses to infer its relationship to *Arabidopsis* GELP clades. The *MS2* candidate protein was analyzed together with the full set of 98 *Arabidopsis* GELP proteins (105 genes reported by Lai et al. (2017) excluding AtGELP25, 29, 31, 34, 57, 77 and 84 because they had lost conserved blocks), and a separate phylogenetic tree was inferred from the resulting alignment. The amino acid sequences were aligned by ClustalX2 [51], and an NJ tree was inferred by MEGA ver. 11 [50].

### Development of a diagnostic marker for candidate gene

To obtain a simple diagnostic marker for the candidate gene (SUGI_0493010), we developed a PCR–RFLP assay targeting the candidate SNP responsible for the MS2-type male sterility in its coding sequence. A primer pair was designed by Primer3Plus [52] to amplify a genomic fragment encompassing this SNP. A suitable restriction enzyme which differentiates between wild and mutant alleles was searched by the REHUNT program [53]. PCR amplification for genotyping the SUGI_0493010 SNP was carried out in a total volume of 10 µL. Each reaction contained approximately 10 ng of genomic DNA, 0.2 µM of each primer (forward: CTTTATCAATGAATCTCCTGGTTT; reverse: AGTAAACCTTCCTGTTGGGTGTC), and 1× QIAGEN Multiplex PCR Master Mix (Qiagen). The cycling program consisted of an initial denaturation at 95 °C for 15 min, followed by 40 cycles of 94 °C for 30 s, 60 °C for 90 s, and 72 °C for 90 s, with a final extension at 72 °C for 10 min. For the PCR–RFLP assay, 5 µL of each PCR product was subjected to restriction digestion in a 10 µL reaction containing 1× restriction buffer and the appropriate restriction enzyme (*Hinf*I) recognizing the SNP site. Digestions were performed at 37 °C overnight. Digested products (5 µL) were separated on 2% agarose gels, stained with ethidium bromide, and visualized under UV illumination to determine the genotype.

## Results

### High-resolution linkage mapping of the *MS2* locus

To refine the localization of the *MS2* gene, we constructed a high-resolution linkage map using the mapping population. Using RNA-Seq data from fertile and sterile individuals, we designed 59 SNP assays corresponding to 41 CJ3006NRE transcript contigs located on the three MS2-linked contigs in the draft genome. Of these, 31 assays derived from 20 genes yielded reliable genotypes and showed linkage to the *MS2* locus, and were therefore incorporated into the refined linkage map (Additional file 2). To relate these draft-genome contigs (ctg668, ctg930, and ctg820) to the chromosome-scale reference, they were aligned to the SUGI_1 assembly, and all three contigs mapped to chromosome 5 (chr5), supporting that the MS2-linked region corresponds to a single locus on chr5 (Additional file 4).

**Fig. 2:**
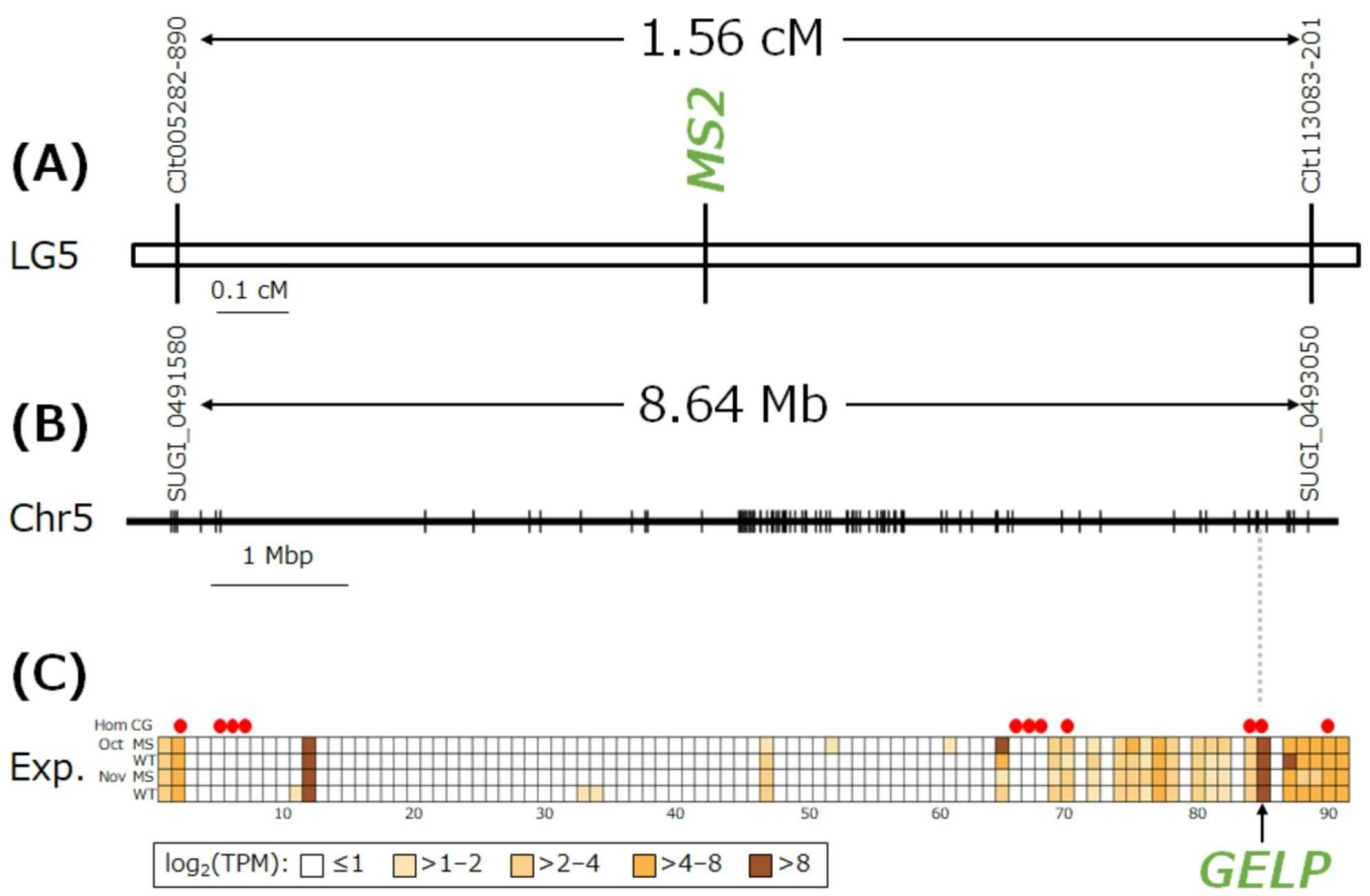
High-resolution linkage mapping of the *MS2* locus and expression profiles of candidate genes. **A** Partial linkage map of linkage group 5 (LG5) showing the *MS2* locus positioned between two flanking markers. **B** The corresponding 8.64 Mb physical interval on chromosome 5 (Chr5), with vertical lines indicating the positions of 91 positional candidate genes. **C** Heatmap showing expression levels (TPM) of the 91 genes in male strobili samples collected in October and November from both fertile (WT) and sterile (MS) individuals. Red dots indicate genes functionally annotated as homologs of *Arabidopsis* genes (Hom CG: homology-based candidate genes) involved in pollen development

The locus was positioned between two flanking markers, CJt005282-890 and CJt113083-201, spanning an 8.64 Mb region on chromosome 5 (Fig. 2, Additional file 2, and Additional file 4). RNA-Seq-derived markers served as anchor markers, bridging the genetic linkage map and the physical genome map, and enabled accurate localization of genes within the *MS2* locus and supporting downstream candidate gene analysis. Within this region, 91 predicted genes from the *standard gene set* (55,246 genes) were identified and used for downstream analysis (Additional file 3).

### Identification of candidate genes related to pollen development

To prioritize functionally relevant candidates, we conducted a homology search against 372 *A. thaliana* genes associated with the GO term “pollen development” (GO:0009555), using the TAIR database. A BLASTX search for the nucleotide sequences of *C. japonica* candidate genes and the *Arabidopsis* gene set revealed that 11 out of the 91 MS2-neighboring genes showed significant sequence similarity to pollen development-related genes (Additional file 3).

### Expression profiling in male strobili

To determine whether the candidate genes were expressed in male reproductive organs, RNA-Seq analysis was performed using male strobili collected in October and November from five fertile and three sterile individuals of the S1-2 family (Additional file 1). An average of 7.4 Gb of RNA-Seq data was obtained per sample. The reads were mapped to the SUGI_1 *permissive gene set*, and transcript abundance was quantified using TPM values. Among the 91 MS2-neighboring genes, 29 genes were expressed (TPM > 1) in at least one of the fertile strobilus samples (Fig. 2 and Additional file 3), and all of these genes were in the SUGI_1 *standard gene set*, ensuring that highly expressed transcripts were not omitted from the expression analysis. Comparison of expression levels between fertile and sterile individuals revealed no statistically significant differences (*p* > 0.01) for these *MS2* candidate genes. This suggests that if *ms2* is caused by a mutation in one of these genes, it likely does not result from transcriptional suppression in sterile individuals.

**Fig. 3:**
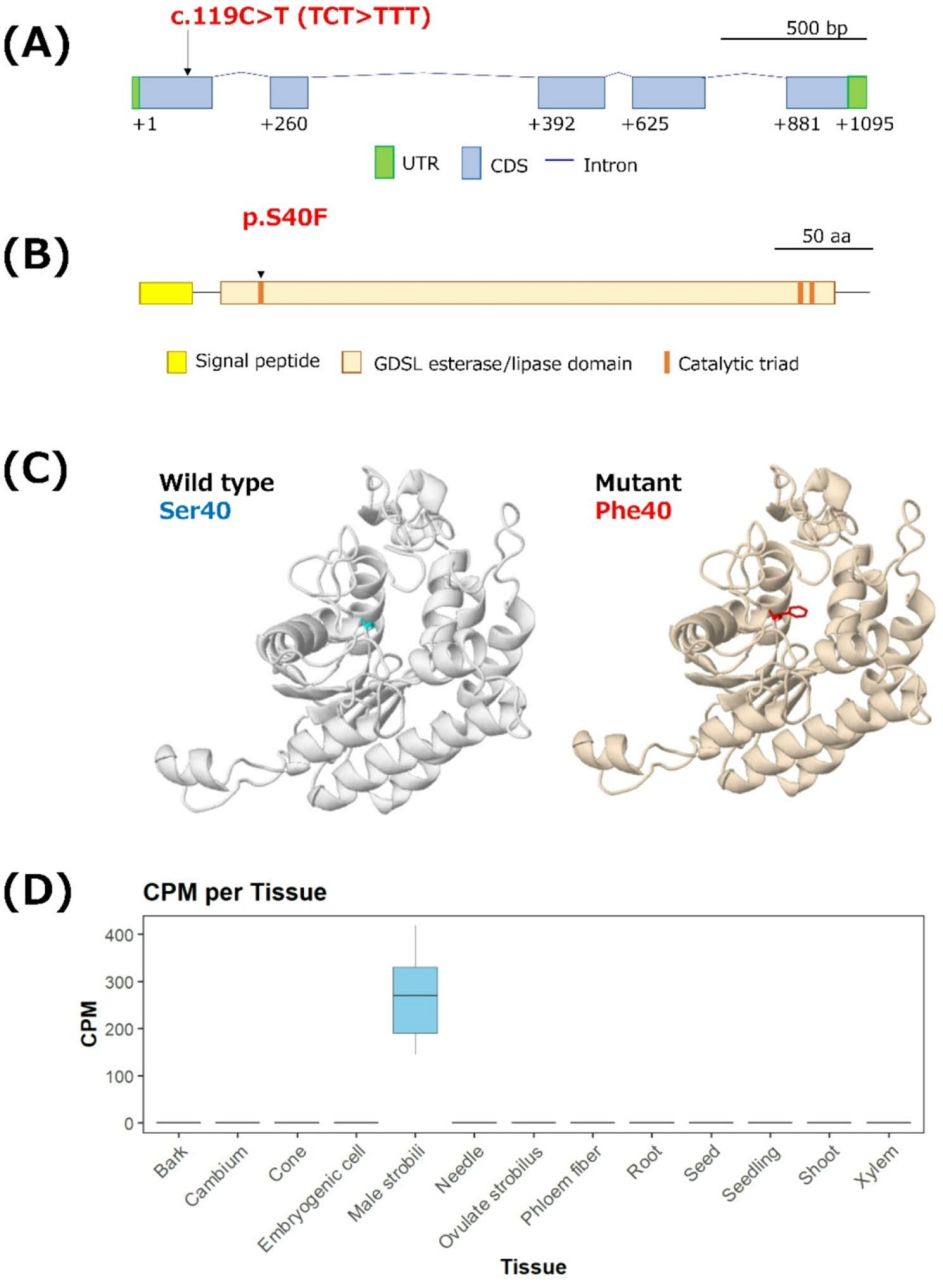
Structure, homology model, and expression profiling of the GELP gene. **A** Exon-intron structure of the GELP gene (SUGI_0493010). Coding sequences (CDS) are shown in blue boxes, untranslated regions (UTRs) in green, and introns as lines connecting exons. Numbers below the exons indicate the relative positions of the CDS nucleotides. The position of the MS2-associated SNP (c.119C>T, TCT>TTT) is indicated with an arrow, corresponding to a missense mutation in the first exon. **B** Schematic representation of the predicted protein encoded by the GELP gene based on InterPro annotation. The signal peptide (yellow), GDSL esterase/lipase domain (light orange), and catalytic triad residues (orange bars) are indicated. The amino acid substitution (p.S40F) caused by the SNP is located within the N-terminal region of the conserved GDSL domain. Only one coding SNP (c.119C>T) leading to the S40F substitution was identified between fertile and sterile haplotypes reconstructed from RNA-Seq data in the S1-2 family. **C** Homology models of the wild-type (left) and S40F mutant (right) GELP proteins generated with Phyre2. The catalytic Ser40 (wild type, cyan) and Phe40 (mutant, red) are highlighted. Ser40 is located within the conserved N-terminal SGNH motif that harbors the catalytic serine in SGNH/GDSL hydrolases and forms part of the canonical Ser–His–Asp catalytic triad. Substitution of Ser40 with the bulkier phenylalanine side chain distorts the geometry of the catalytic pocket and reduces the modeled cavity volume, thereby decreasing the predicted accessibility of fatty-acid substrates. **D** Expression profile of GELP across 13 tissues of *C. japonica* based on RNA-Seq data from SugiExDB [42]. Counts per million (CPM) values are shown as boxplots. GELP exhibits strong and specific expression in male strobili, with no detectable expression in other tissues, including needles, cambium, roots, and ovulate strobili

### Detection and validation of deleterious mutations

We next examined whether deleterious mutations were present in the coding regions of the 29 expressed genes. Variant calling was conducted using RNA-Seq data, and amino acid substitutions were evaluated using PROVEAN to assess their functional impact. Three deleterious amino acid substitutions (PROVEAN score ≤ −2.5) were identified in two genes (Additional file 3). Notably, one gene (CJt112073 or SUGI_0493010), encoding a GDSL-type esterase/lipase (GELP), contained a substitution from serine to phenylalanine at position 40 (p.S40F) located within the predicted enzymatic active site (Fig. 3). Structural modeling of this candidate protein using Phyre2, followed by assessment with Missense3D, showed that substitution of Ser40 with a bulky phenylalanine distorts the active-site pocket (Fig. 3), breaking buried H-bond and reducing the catalytic cavity volume by approximately 85 Å³ relative to the wild-type model, consistent with reduced accessibility of fatty-acid substrates. The other two mutations were found in a gene homologous to *Arabidopsis INTS1* (SUGI_0492850), which is not known to be involved in pollen development and did not meet the homology-based candidate criteria (Additional file 3).

To validate the presence of the *GELP* mutation in another genetic background, we examined `Gosenshi-1,` an individual previously confirmed to carry the *MS2* mutation in a heterozygous state (*Ms2*/*ms2*) through controlled crossing experiments [54]. RNA-Seq-based analysis confirmed that the same S40F substitution was present in `Gosenshi-1` (Additional file 5). The consistent presence of this mutation in both *ms2*/*ms2* and *Ms2*/*ms2* individuals supports the hypothesis that *GELP* is the most likely candidate gene for MS2-type male sterility in *C. japonica*. Furthermore, RNA-Seq analysis showed that the *INTS1* mutation was absent in `Gosenshi-1` (Additional file 5), confirming this gene to be excluded from the list of mutational candidates.

### Structural, functional, expressional, and phylogenetic profiling of GELP

Structural annotation of the *GELP* gene (SUGI_0493010) revealed five exons and four introns (Fig. 3A). TSSPlant predicted multiple transcription start sites (TSSs) within the upstream region of the gene which exceeded the prediction threshold. Among these, one TSS candidate was fully supported by the full-length cDNA sequence obtained by biotinylated CAP trapper method [38]; this site was therefore considered the most likely transcription initiation site. The 5′ untranslated region (UTR) was defined as the 19-bp region between this TSS and the start codon. In contrast, Iso-Seq reads aligned one base upstream of the predicted TSS, likely due to non-templated nucleotide addition by the reverse transcriptase during cDNA synthesis, which can introduce a non-templated nucleotide at the 5′ end [55]. Taken together, these observations support the accuracy of the TSS identified by TSSPlant and the full-length cDNA.

The 3′ end of the transcript was inferred based on the termination pattern of Iso-Seq reads downstream of the stop codon. The majority of reads converged at a position where read depth reached a local minimum, approximately 28 bp downstream of a canonical polyadenylation (polyA) signal (AATAAA), and was followed by a plateau in coverage. This position likely represents the major polyadenylation cleavage site. Additionally, a minor cleavage site was suggested further downstream, approximately 26 bp beyond a secondary polyA signal.

Functional annotation of the predicted protein sequence (364 amino acids) identified a conserved GDSL esterase/lipase domain (PF00657), belonging to the SGNH hydrolase superfamily (IPR036514). The catalytic triad—Ser40, Asp329, and His332—was conserved, and the S40F missense mutation identified in *ms2/ms2* individuals lies within this catalytic region (Fig. 3B). Notably, the affected serine (S40) is not only a part of the predicted catalytic triad (S40, D329, H332) but also corresponds to the conserved serine residue within the GDSL motif (Gly–Asp–Ser–Leu), which defines this esterase/lipase family. Substitution of this critical serine to phenylalanine (S40F) is therefore expected to abolish enzymatic activity. To confirm the specificity of this mutation, haplotype sequences spanning the GELP coding region were reconstructed from RNA-Seq data for both fertile and sterile individuals in the S1-2 family. Among all identified variants, only a single nucleotide substitution (c.119 C > T) corresponding to the S40F amino acid change was found, indicating that this mutation is the sole coding difference between the two haplotypes. These findings strongly support the role of SUGI_0493010 as the candidate gene underlying MS2-type male sterility.

In addition to the structural and functional annotations, tissue-specific expression profiles were examined using RNA-Seq data from 13 *C. japonica* tissues available in SugiExDB [42]. CPM values were visually compared across tissues to explore tissue-preferential expression tendencies. GELP exhibited strong and specific expression in male strobili, with no detectable expression in other tissues, including needles, cambium, roots, and ovulate strobili (Fig. 3D, and Additional file 6). This expression pattern supports the involvement of GELP in male reproductive development.

We further examined the temporal expression of SUGI_0493010 in male strobili using the microarray dataset of [15] and found that its expression peaked at intermediate developmental stages corresponding to late October to early November, with much lower expression at earlier and later stages (Additional file 7). Thus, GELP expression is not only highly specific to male strobili but is also confined to a distinct developmental window during microspore development.

To avoid biases arising from variable N- and C-terminal regions, phylogenetic inference for the *C. japonica* GELP family was performed using only the Pfam Lipase_GDSL (PF00657)-aligned domain regions extracted from HMMER domtblout coordinates. HMMER searches against the Pfam-A database identified PF00657 hits in 191 proteins from a comprehensive list of 209 GELP candidates (Additional file 8). After excluding truncated matches, 162 PF00657 domain sequences were retained for multiple sequence alignment. Subsequent tree inference resolved multiple GELP lineages within *C. japonica*, enabling placement of the *MS2* candidate gene (SUGI_0493010) relative to other PF00657-supported paralogs (Additional file 9).

Phylogenetic analysis based on a neighbor-joining tree of full-length *MS2* candidate GELP amino-acid sequences from *C. japonica* and 98 *Arabidopsis* GELPs [56] placed SUGI_0493010 within subclade IIc of clade II (Additional file 9), where it grouped most closely with AtGELP98 (AT5G41890).

### Development of a diagnostic marker for candidate gene

**Fig. 4:**
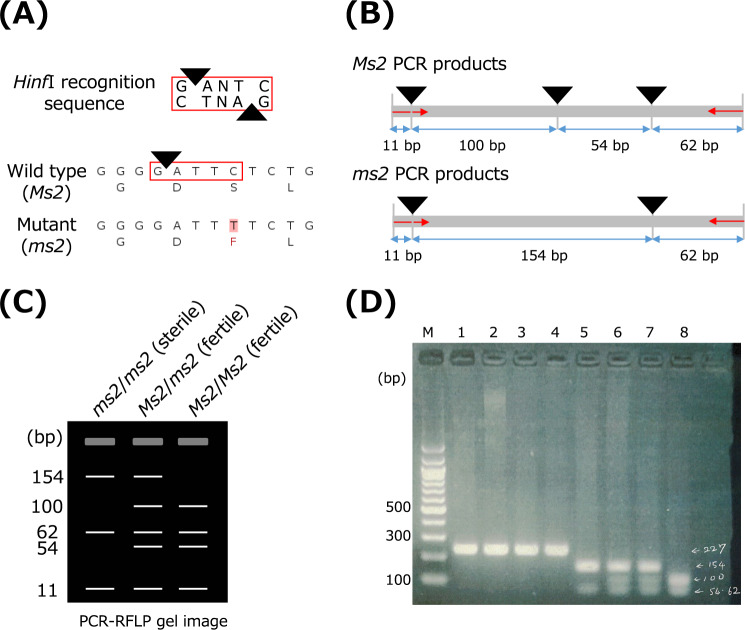
Development and validation of a PCR-RFLP marker for the MS2-associated GELP SNP. **A** Schematic representation of the *Hinf*I recognition sequence and the nucleotide and amino acid changes at the MS2-associated SNP in SUGI_0493010. Closed triangles indicate HinfI cleavage sites. In the wild-type *Ms2* allele, the codon encodes the conserved GDSL motif and forms a complete *Hinf*I site, whereas in the mutant *ms2* allele the c.119C>T substitution disrupts the restriction site and introduces the S40F substitution. **B** Schematic diagram of the PCR amplicon and *Hinf*I restriction sites. Red arrows indicate the forward and reverse primers, and closed triangles indicate the positions of *Hinf*I cleavage sites within the amplicon, including a site located inside the forward primer. **C** Idealized gel image illustrating the expected PCR-RFLP banding patterns for the three genotypes at the *MS2* locus (*ms2*/*ms2*, *Ms2*/*ms2*, and *Ms2*/*Ms2*). **D** Agarose gel electrophoresis of the PCR-RFLP assay. Lane M, size marker; lanes 1–4, undigested PCR products from `Shindai-1` (*ms2*/*ms2*), `S1NK4` (*Ms2*/*ms2*), `Gosenshi-1` (*Ms2*/*ms2*), and `Nakakubiki-4` (*Ms2*/*Ms2*), respectively; lanes 5–8, corresponding samples after *Hinf*I digestion

The PCR–RFLP assay developed for *MS2* was successfully used to discriminate individual genotypes (Fig. 4). The PCR amplified a 227-bp fragment from both the wild-type and mutant alleles. The PCR products were then digested into smaller fragments (154, 100, 62, 54, and 11 bp). At the candidate SNP site (c.119 C > T), the mutant (sterile) allele was not digested, whereas the wild-type allele was cleaved by the restriction enzyme. In addition, *Hinf*I has two additional recognition sites within the amplicon, generating the 11- and 62-bp fragments. When applied to the mapping population, the PCR–RFLP genotypes showed complete co-segregation with the fertile vs. sterile phenotypes: all male-sterile individuals carried the homozygous mutant (*ms2*/*ms2*) pattern, whereas all male-fertile individuals were heterozygous (*Ms2*/*ms2*). No recombinants were observed in this population, indicating that the genetic distance between the SUGI_0493010 SNP and the *MS2* locus is effectively 0.0 cM. The heterozygous `Gosenshi-1` also exhibited the expected heterozygous restriction pattern, consistent with its *Ms2*/*ms2* genotype inferred from segregation analysis.

## Discussion

In this study, we identified a strong candidate gene for the MS2-type male sterility in *Cryptomeria japonica* through a comprehensive approach that combined genetic linkage mapping, gene expression analysis, and functional prediction of amino acid substitutions. Our results suggest that a GDSL-type esterase/lipase gene (*GELP*), which harbors a deleterious amino acid substitution, is the most likely candidate gene for MS2-type male sterility (Additional file 10).

The candidate *GELP* gene resides within the 1.56 cM interval surrounding the *MS2* locus on linkage group 5, corresponding to an 8.64 Mb region of the reference genome. Among the 91 genes in this interval, *GELP* was one of 11 genes with homology to *Arabidopsis* genes involved in pollen development and one of 29 genes expressed in male strobili. Expression analysis showed that *GELP* is specifically expressed in male strobili (Fig. 3D), and is present in both fertile and sterile individuals (Fig. 2). In addition, the candidate GELP gene also showed a transient expression peak at the intermediate stages of male strobilus development (Additional file 7), further linking its activity to a specific window of microspore development. This supports the idea that the sterility phenotype arises not from a lack of expression, but from a functional disruption caused by the amino acid substitution. Notably, the gene exhibited a serine-to-phenylalanine substitution (S40F) within a predicted active site (Fig. 3), which was predicted by PROVEAN and structural analysis with Missense3D to be functionally deleterious. The affected serine residue is part of the conserved catalytic triad of GDSL esterases/lipases. This serine hydroxyl group is essential for nucleophilic attack during catalysis, and its substitution with a bulky, nonpolar phenylalanine is expected to abolish enzymatic activity. Consistent with this expectation, site-directed mutagenesis studies for GDSL/SGNH hydrolases have shown that replacement of the catalytic Ser in block I with non-nucleophilic residues (Ser to Ala) leads to complete loss of hydrolase or transferase activity [57]. This body of biochemical evidence [58] strongly supports the conclusion that the S40F mutation causes a loss of catalytic function in the *C. japonica* GELP, consistent with the *MS2* phenotype. Furthermore, the same mutation was identified (Additional file 5) in a heterozygous (*Ms2*/*ms2*) individual, `Gosenshi-1,` which was derived from an independent genetic background, indicating that this mutation is consistently associated with MS2-type male sterility in *C. japonica*.

GDSL esterases/lipases have been implicated in a variety of biological processes, including lipid metabolism, cuticle formation, and reproductive development in plants [59–62]. A genome-wide phylogenetic analysis in *Arabidopsis thaliana* grouped 98 GELP genes into four major clades (I–IV) and several subclades [56]. When we reconstructed a neighbor-joining tree using the full-length amino acid sequences of these 98 *Arabidopsis* GELPs together with *C. japonica* GELP proteins, SUGI_0493010 clustered within subclade IIc of clade II (Additional file 9). Lai et al. (2017) reported that clade II contains several GELPs involved in abiotic stress responses (e.g. LiCl or high-glucose stress and wound responses) and in pollination, including AtGELP83 (CDEF1) and JNP1, which influence pollen hydration and pollen-tube penetration [56]. This phylogenetic placement suggests that SUGI_0493010 may share functional themes with these clade II GELPs, such as modification of extracellular or cell-wall components at the male gametophyte–sporophyte interface, although its precise biochemical function remains to be determined. Within subclade IIc, SUGI_0493010 grouped most closely with AtGELP98 (AT5G41890). According to a tissue-resolved RNA-seq atlas for *Arabidopsis* [63], AT5G41890 shows strongly enriched expression in anthers and floral organs with little or no expression detected in vegetative tissues (Additional file 11). This expression pattern is reminiscent of the male-strobilus-specific expression of SUGI_0493010 in *C. japonica* (Fig. 3D), suggesting that these genes may share conserved roles in male reproductive tissues, although the biochemical function of AtGELP98 has not yet been characterized.

In *Arabidopsis thaliana*, mutation of the GDSL esterase/lipase gene has been shown to cause male sterility due to defects in pollen wall formation [64]. Given the role of sporopollenin and lipids in pollen wall structure, it is plausible that the *GELP* gene in sugi plays a similar role in pollen development. Supporting this hypothesis, several morphological characteristics observed in *ms2* mutant individuals are consistent with those reported in *A. thaliana*. Loss-of-function mutations in this GDSL gene resulted in abnormal pollen wall formation, irregularly shaped microspores, early microspore degeneration, and partial or complete nuclear loss [62]. Notably, clumping or high-density aggregation of microspores—likely due to defects in post-meiotic microspore separation—has also been observed in MS2-type male sterility in *C. japonica*, where densely packed and irregular microspores, some lacking nuclei, are found [9, 10]. The predicted loss of catalytic activity in SUGI_0493010 may impair specific lipid or ester remodeling steps necessary for microspore release, leading to persistent adhesion between sister microspores. Defective remodeling of the extracellular matrix around tetrads could thus plausibly result in the characteristic tetrad aggregation observed in MS2-type male sterility. These shared phenotypes across species strongly support the idea that GELP plays a conserved and essential role in male gametophyte development, likely through contributing to proper microspore maturation.

In *C. japonica*, our *MS2* candidate *GELP* can also be placed in the context of other cloned male-sterility genes. *MS1* is encoded by CJt020762 (SUGI_0915590) [17, 28, 65], a lipid transfer protein gene in which frame-shift deletions cause a loss of function and lead to microspore collapse after tetrad separation, while *MS4* is encoded by *CjTKPR1* [18], a TETRAKETIDE α-PYRONE REDUCTASE 1 involved in sporopollenin biosynthesis, where a single-nucleotide substitution abolishes pollen production. In contrast, *MS2* is associated with a missense mutation in the catalytic serine of a GELP, and *ms2* mutants exhibit microspore clumping and defective pollen separation after otherwise normal microsporogenesis [10]. Thus, at least three distinct lipid-associated proteins—an LTP (MS1), a GDSL esterase/lipase (MS2), and TKPR1 (MS4)—contribute to male sterility at overlapping but distinct steps of pollen wall and microspore development in *C. japonica*. Future work will be needed to clarify whether these loci act in a common pathway or represent parallel, partially redundant routes to male sterility.

Independent genetic evidence for SUGI_0493010 as the *MS2* candidate gene has recently become available [66], where a second mutant allele, *ms2-2* (c.116_123delATTCTCTG), in the same GELP gene in `Fukushima-funen 3` was reported. This allele causes a frameshift and a premature stop codon near the N-terminus of the protein, and an individual homozygous for *ms2-2* was shown to be male sterile. Together with the originally identified *ms2-1* missense mutation (S40F), these findings establish an allelic series at the same locus and substantially strengthen the genetic support for SUGI_0493010 as the gene underlying MS2-type male sterility in *C. japonica*. However, as discussed later, strong genetic association and independent allelic evidence are not fully equivalent to direct functional validation in the species itself.

Despite this collective evidence supporting *GELP* as the candidate gene, several questions remain to be resolved. Although the *MS2* allele generally confers an almost complete absence of pollen in homozygous *ms2*/*ms2* individuals, we occasionally observed anthers that contained a small number of pollen grains (Additional file 12), indicating that the *ms2*/*ms2* genotype does not always result in absolute pollen abortion [10]. At present, the mechanisms underlying such residual pollen production are unclear. One potential explanation is functional overlap with other genes, such as the neighboring SUGI_0493000, which encodes another GDSL esterase/lipase protein and is located adjacent to GELP (SUGI_0493010). However, SUGI_0493000 is expressed in male strobili of both fertile and sterile individuals, its TPM levels do not differ between the two groups, and no deleterious amino-acid substitution was detected in this gene, suggesting that any compensatory effect, if present, is unlikely. More broadly, our genome-wide survey identified 119 GDSL esterase/lipase genes that harbor a Pfam GDSL domain and show detectable expression in male strobili (Additional file 8), but whether any of these genes contribute to sporadic residual pollen production remains unknown.

Additional factors may contribute to variation in phenotypic expressivity among *ms2/ms2* individuals. One possibility is regulatory variation at other pollen-related loci, including additional GELP genes expressed in male strobili. Such variation could involve epigenetic mechanisms or differences in transcriptional control, although the present study does not directly test these possibilities [67, 68]. Such regulatory variation, if present, could alter the timing and/or cell-type specificity of GELP expression during the narrow developmental window when microspore separation occurs. Such effects may not be fully captured by bulk RNA-seq, which averages signals across heterogeneous tissues and closely spaced developmental stages [69].

Furthermore, the segregation ratio of fertile and sterile individuals in the S1-2 family deviated from the expected 1:1 Mendelian ratio. This may reflect viability effects or segregation distortion related to the *MS2* locus. Possible explanations include impaired pollen function and/or reduced viability during early *ms2*/*ms2* embryo development. However, the present data are insufficient to distinguish among these possibilities. Importantly, within the set of reliably phenotyped individuals used for mapping, the *MS2* phenotype co-segregated perfectly with the SUGI_0493010 mutation (Additional file 2), consistent with the present mapping result, although validation in larger populations remains necessary.

A limitation of this study is that the identification of SUGI_0493010 as the *MS2* gene remains based on positional and correlative evidence rather than direct functional proof. In our BC-type mapping population, we deliberately prioritized phenotypic certainty over sample size, because misclassification of even a single individual for this essentially all-or-none trait could create an apparent recombinant and alter local marker order. As a result, the attainable mapping resolution was inherently constrained by the number of informative meiotic events represented by the 95 confidently phenotyped individuals analyzed here. Accordingly, although the *MS2* locus was narrowed to a relatively short genetic interval of 1.56 cM, this still corresponded to an 8.64 Mb physical region. This likely reflects both the limited number of recombination events captured in the mapping population of 95 offspring and heterogeneity in recombination along chromosome 5 [25]. SUGI_0493010 is strongly supported as the leading candidate by complete co-segregation within the analyzed material, male-strobilus-specific expression, functional annotation, and the predicted deleterious effect of the S40F substitution. However, direct functional evidence demonstrating that this *GELP* gene causes the *MS2* phenotype is still lacking. Future work should therefore focus on direct functional validation of this gene, for example by (i) introducing targeted mutations in the *GELP* locus using genome editing [70, 71], (ii) testing whether expression of the wild-type allele can complement male-sterile phenotypes in suitable heterologous systems [18], and/or (iii) performing *in vitro* biochemical assays to compare the enzymatic properties of the wild-type and mutant GELP proteins [57]. Such experiments will be essential to conclusively demonstrate causality and to clarify the precise biochemical role of this GELP in microspore development. In this respect, the most decisive evidence would be obtained from recreating or repairing the mutation in sugi itself, as genome editing has already provided direct native-species validation for pollen-development genes in *C. japonica* [70]. By contrast, heterologous complementation in another species [18] and biochemical assays using recombinant proteins would provide important orthogonal support, but such evidence would still remain indirect with respect to the native sugi phenotype. This distinction is particularly relevant for GDSL esterase/lipase proteins, which constitute a functionally diverse enzyme family with broad and often difficult-to-predict substrate specificities [72]. Therefore, positive results from heterologous or *in vitro* systems would reinforce the candidacy of SUGI_0493010, whereas negative results would be more difficult to interpret and would not necessarily exclude this gene, because failure to rescue a phenotype or detect enzymatic activity may reflect missing post-translational processing or other assay-specific constraints rather than lack of biological function [73, 74]. Nevertheless, these approaches remain highly valuable for refining mechanistic hypotheses and prioritizing subsequent direct validation in *C. japonica*.

## Conclusions

In this study, we identified a strong candidate gene for *MALE STERILITY 2* (*MS2*) in *Cryptomeria japonica* using a map-based approach that integrated high-resolution linkage mapping, transcriptome profiling, and mutation analysis. The *MS2* locus was localized to an 8.64 Mb region on chromosome 5, within which a GDSL-type esterase/lipase gene (*GELP*) was identified as the most likely candidate gene. A deleterious amino acid substitution (S40F) was detected in sterile individuals and validated in an independent heterozygous line, supporting the association between the mutation and the *MS2* sterility phenotype. These findings provide a key genetic resource for breeding pollen-free *C. japonica* trees, which are of increasing social importance in the context of allergenic pollen mitigation. Moreover, the identification of *GELP* as a candidate gene adds to our understanding of the molecular mechanisms underlying male sterility in conifers and highlights the utility of integrative genomic approaches in forest tree research.

## Supplementary Information

Supplementary Material 1. Supplementary Table 1. Summary of RNA-Seq samples and sequencing information.

Supplementary Material 2.Supplementary Table 2. Details of markers and linkage map refinement around the *MS2* locus.

Supplementary Material 3.Supplementary Table 3 List of genes located on *MS2* locus, and their characteristics.

Supplementary Material 4.Supplementary Figure 1. Linkage and physical maps around the *MS2* locus. Description of data: Top: Linkage map of LG5 around the *MS2* locus reported by Hasegawa *et al*. (2018), showing Axiom SNP markers and the corresponding CJ3006NRE transcript IDs in parentheses. Middle: Refined linkage map of LG5 obtained in this study. The *MS2* region is highlighted in yellow and is delimited by the flanking markers CJt005282-890 and CJt113083-201 with 1.56 cM interval. Bottom: Schematic correspondence between the MS2-linked draft-genome contigs (ctg668, ctg930, and ctg820; Fujino *et al*., draft genome ver. 0.1, unpublished) and the chromosome-scale reference assembly SUGI_1 (Fujino *et al*., 2024). The physical *MS2* region on chr5 is shown in yellow (280.6–289.3 Mbp), and includes the *GELP* candidate gene (SUGI_0493010) and its neighboring genes SUGI_0491580 and SUGI_0493050. Genes shown in red are common between Hasegawa *et al*. (2018) and this study. The yellow interval corresponds to the *MS2* region shown in Figure 2.

Supplementary Material 5.Supplementary Figure 2. Amino-acid sequence alignment of the *MS2* mutational candidate genes in *Cryptomeria japonica*: (A) INTS1 and (B) GELP. Description of data: Alignment of the amino acid sequences of SUGI *standard gene set* (SUGI_0492850 and SUGI_0493010 for INTS1 and GELP, respectively), Gosenshi-1_a1 (wild-type allele), Gosenshi-1_a2 (mutant allele), and the ms2 mutant from the S1-2 family, generated in CLC Genomics Workbench ver. 20.0.4 (Qiagen). Residues are colored according to side‐chain polarity. Red arrows indicate positions of deleterious amino acid substitutions predicted by PROVEAN (score ≤ –2.5). For INTS1, two deleterious amino acid substitutions (at positions 613 and 1047) were identified in the S1-2 family, but these substitutions were absent in `Gosenshi-1.` For GELP, two substitutions are observed relative to SUGI_0493010 (S40F and C352Y), but only S40F is deleterious and uniquely distinguishes the *ms2* mutant from the wild-type allele. In addition, the 352nd residue in `Gosenshi-1` (*Ms2*/*ms2*) is homozygous for tyrosine (Y/Y), as indicated by the blue arrow, further indicating that C352Y is unlikely to be the causal mutation.

Supplementary Material 6.Supplementary Table 4. CPM values of the GELP candidate gene (SUGI_0493010) across 13 tissues of *Cryptomeria japonica*. Description of data: Each row corresponds to one RNA-Seq library in SugiExDB. "Sample ID" indicates the SugiExDB library sample identifier, "Tissue" denotes the assigned tissue type, and "CPM" gives the counts-per-million value for the GELP candidate gene (SUGI_0493010). Replicate libraries for the same sample are listed separately.

Supplementary Material 7.Supplementary Figure 3. Temporal expression profile of SUGI_0493010 in male strobili based on the microarray dataset. Description of data: Normalized signal intensities of microarray probes matching SUGI_0493010 are shown across ten developmental stages of male strobili (mean ± SD). Developmental stages are defined according to Tsubomura et al. (2016) as follows: Stage 1, appearance of scale primordia in the axils; Stage 2, differentiation of microsporangia after scale formation; Stage 3, recognizable microsporangial wall, middle layer, tapetum and pollen mother cells; Stage 4, entry of pollen mother cells into meiosis; Stage 5, completion of meiosis and tetrad formation; Stage 6, degeneration of the callose wall and release of microspores into the microsporangium; Stage 7, degeneration of the tapetum; Stage 8, formation of fibrous bands in the microsporangial wall; Stage 9, pollen mitotic division and maturation of pollen grains; Stage 10, anther dehiscence and pollen release. All probes show a pronounced expression peak at intermediate stages (around Stages 5–7), corresponding to the period of tetrad formation, microspore release, and tapetum degeneration.

Supplementary Material 8.Supplementary Table 5. Genome-wide annotation and expression profiles of GDSL esterase/lipase (GELP) genes in *Cryptomeria japonica* male strobiliDescription of data: A comprehensive list of 209 GELP candidates is provided together with Pfam-A/HMMER evidence for GDSL esterase/lipase-related domains (including Lipase_GDSL (PF00657.24) and related Pfam entries), and PF00657-specific domtblout-derived coordinates (alifrom–alito) used to extract the PF00657-aligned domain regions for downstream multiple sequence alignment and phylogenetic analysis. PF00657 hits were detected in 191 proteins, and after excluding truncated matches, 162 PF00657 domain sequences were retained for alignment and neighbor-joining tree inference. The table also includes transcript abundance values (TPM) in male strobili for all RNA-Seq samples analyzed in this study, together with the summary column “TPM max. in WT,” which indicates the maximum TPM value among wild-type male strobilus samples. The binary flag “is_GELP_PFAM_any” indicates whether a gene has GDSL-related Pfam support. In the Discussion, the subset referred to as GELP genes with a Pfam GDSL domain and detectable expression in male strobili corresponds to entries with is_GELP_PFAM_any = 1 and TPM max. in WT > 1. Gene-based functional annotations (Araport11 best hits, UniProt and EggNOG information, and predicted protein domains) were extracted from the publicly available ForestGEN annotation dataset for the SUGI_1 reference genome (https://forestgen.ffpri.go.jp/en/info_sugi1.html).

Supplementary Material 9.Supplementary Figure 4. Phylogenetic placement of the MS2-candidate GELP (SUGI_0493010) among GELP proteins in *Cryptomeria japonica* and *Arabidopsis thaliana*. Description of data: (A) Neighbor-joining tree of 162 putative GELP proteins from *Cryptomeria japonica*, inferred from an amino-acid alignment of the region corresponding to the conserved GDSL esterase/lipase domain. The MS2-candidate GELP, SUGI_0493010 (CjGELP), is indicated by a blue star. (B) Neighbor-joining tree showing the phylogenetic relationships between CjGELP (SUGI_0493010, blue star) and 98 Arabidopsis GELP proteins (105 genes reported in Lai *et al*. (2017), excluding AtGELP25, 29, 31, 34, 57, 77 and 84), constructed from full-length amino-acid sequences. Colored arcs delineate the four major clades (I–IV) and the subclades within clade II (IIa–IIc) defined by Lai *et al*. (2017). CjGELP (SUGI_0493010) falls within subclade IIc.

Supplementary Material 10.Supplementary Figure 5. Summary of the candidate gene (CG) screening process, illustrated as a Venn diagram. Description of data: A total of 91 positional candidate genes (Pos. CG) were initially identified based on genome sequence and linkage analysis. Among these, 11 genes were functionally annotated as homologs of Arabidopsis genes involved in pollen development (Homology-based candidate genes; Hom. CG), 29 genes were expressed in male strobili based on RNA-Seq data (Expressional candidate genes; Exp. CG), and 2 genes contained predicted deleterious mutations (Mutational candidate genes; Mut. CG). Only one gene, a GDSL esterase/lipase protein (GELP), met all three criteria and was thus identified as the most likely candidate gene responsible for MS2-type male sterility in *C. japonica*. Note: One of the two Mutational CGs (homologous to *Arabidopsis* INST1) does not fall into the Hom. CG category.

Supplementary Material 11.Supplementary Figure 6. Tissue-specific expression pattern of AT5G41890 in *Arabidopsis thaliana* based on the RNA-seq atlas. Description of data: Boxplots showing RPKM values of AT5G41890 across multiple tissues in *Arabidopsis thaliana*, derived from the RNA-seq atlas (Klepikova *et al*. 2016). For each tissue, all atlas samples annotated with that tissue are summarized; boxes indicate the interquartile range of observed RPKM values. AT5G41890, which represents the closest *Arabidopsis* GELP homolog to SUGI_0493010 in the phylogenetic analysis, exhibits strongly enriched expression in reproductive tissues, particularly anthers and floral organs, whereas expression is low or undetectable in most vegetative tissues. This reproductive-tissue–biased expression pattern parallels the male-strobilus-specific expression of SUGI_0493010 in *Cryptomeria japonica*.

Supplementary Material 12.Supplementary Figure 7. Microscopic cross-section of a male strobilus from a sterile individual (`Y677`) in S1-2 family. Description of data: A partial formation of pollen was observed in the male strobilus of an individual with the *ms2*/*ms2* genotype. The sample was collected from the Chiyoda nursery on 20 December 2023. The scale bar is based on an approximate size of the male strobilus (~4 mm) and is provided as a reference only.

## Data Availability

The RNA-Seq Illumina reads were deposited in the Sequence Read Archive database of the DNA Data Bank of Japan (DDBJ) under accession number DRR733425–DRR733440. All additional datasets and materials used during the current study are available from the corresponding author upon reasonable request.

## Abbreviations

CDS: Coding Sequence
DEG: Differentially Expressed Gene
GELP: GDSL esterase/lipase protein
GO: Gene Ontology
INTS1: Integrator Complex Subunit 1
kb: Kilobase pairs
LG: Linkage Group
Mb: Megabase pairs
MS: Male Sterility
QTL: Quantitative Trait Locus
RNA-Seq: RNA Sequencing
SNP: Single Nucleotide Polymorphism
TPM: Transcripts Per Million
TSS: Transcription Start Site
UTR: Untranslated Region
