# Supplementary figures and images for: Positional identification of a candidate gene for MALE STERILITY 2 (MS2) by linkage mapping and transcriptomic data in Cryptomeria japonica D. Don
Source: BMC Genomics. 2026 May 19;27:595. doi: 10.1186/s12864-026-12907-4 (PMC13339982; doi:10.1186/s12864-026-12907-4)

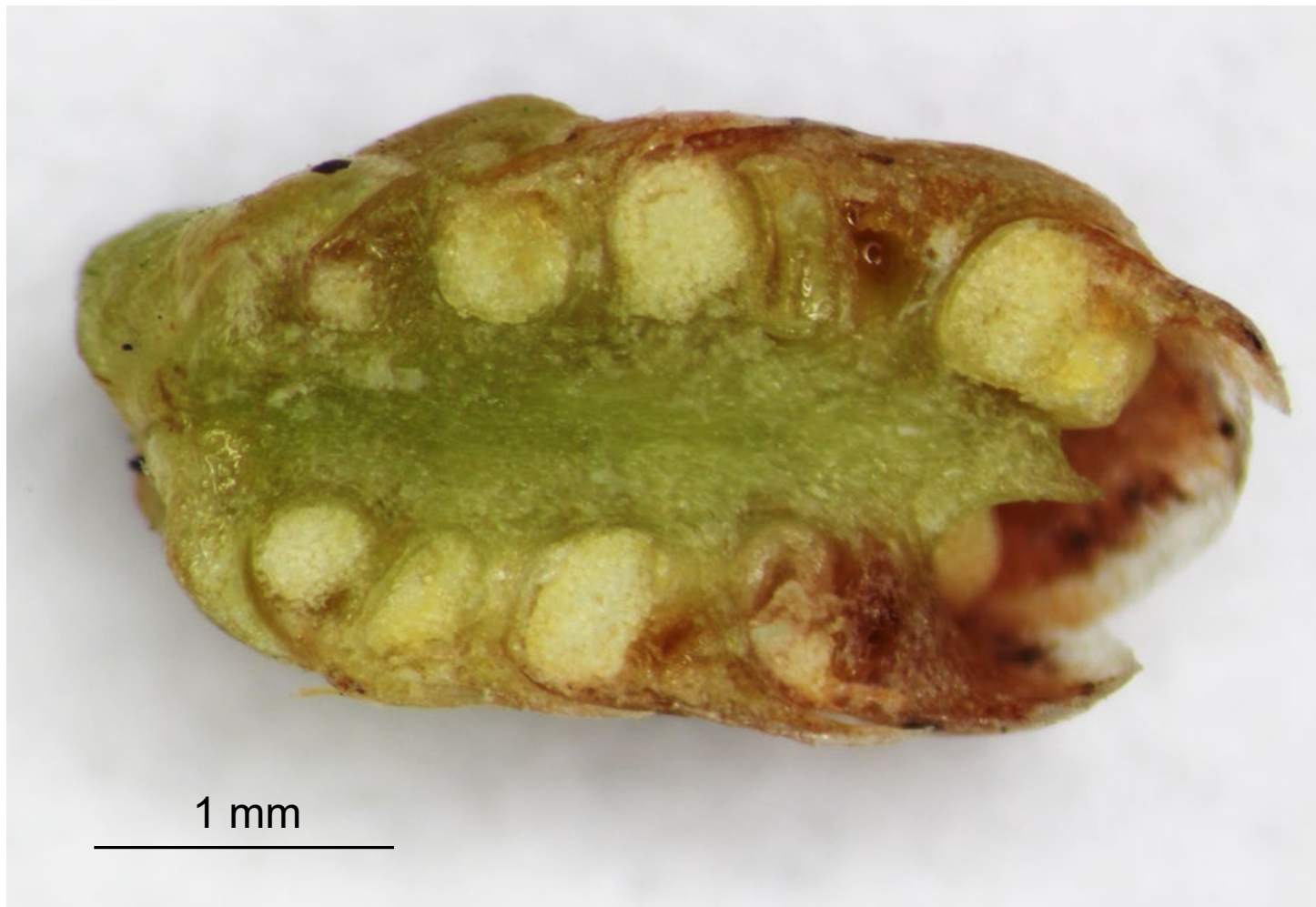

Supplement: Supplementary file 2 — Supplementary Material 2.Supplementary Table 2. Details of markers and linkage map refinement around the MS2 locus. [file 12864_2026_12907_MOESM12_ESM.pdf]

SUGI\_1 standard gene set : 55,246 genes

Pos. CG : 91

Hom. CG: 11

Exp. CG: 29

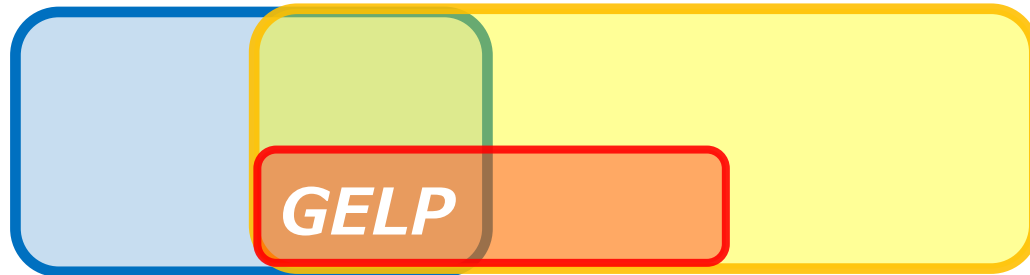

Mut. CG: 2

Supplement: Supplementary file 5 — Supplementary Material 5.Supplementary Figure 2. Amino-acid sequence alignment of the MS2 mutational candidate genes in Cryptomeria japonica: (A) INTS1 and (B) GELP. Description of data: Alignment of the amino acid sequences of SUGI standard gene set (SUGI_0492850 and SUGI_0493010 for INTS1 and GELP, respectively), Gosenshi-1_a1 (wild-type allele), Gosenshi-1_a2 (mutant allele), and the ms2 mutant from the S1-2 family, generated in CLC Genomics Workbench ver. 20.0.4 (Qiagen). Residues are colored according to side‐chain polarity. Red arrows indicate positions of deleterious amino acid substitutions predicted by PROVEAN (score ≤ –2.5). For INTS1, two deleterious amino acid substitutions (at positions 613 and 1047) were identified in the S1-2 family, but these substitutions were absent in `Gosenshi-1.` For GELP, two substitutions are observed relative to SUGI_0493010 (S40F and C352Y), but only S40F is deleterious and uniquely distinguishes the ms2 mutant from the wild-type allele. In addition, the 352nd residue in `Gosenshi-1` (Ms2/ms2) is homozygous for tyrosine (Y/Y), as indicated by the blue arrow, further indicating that C352Y is unlikely to be the causal mutation. [file 12864_2026_12907_MOESM10_ESM.pdf]

Expression pattern of GELP-matching probes across male strobili stages

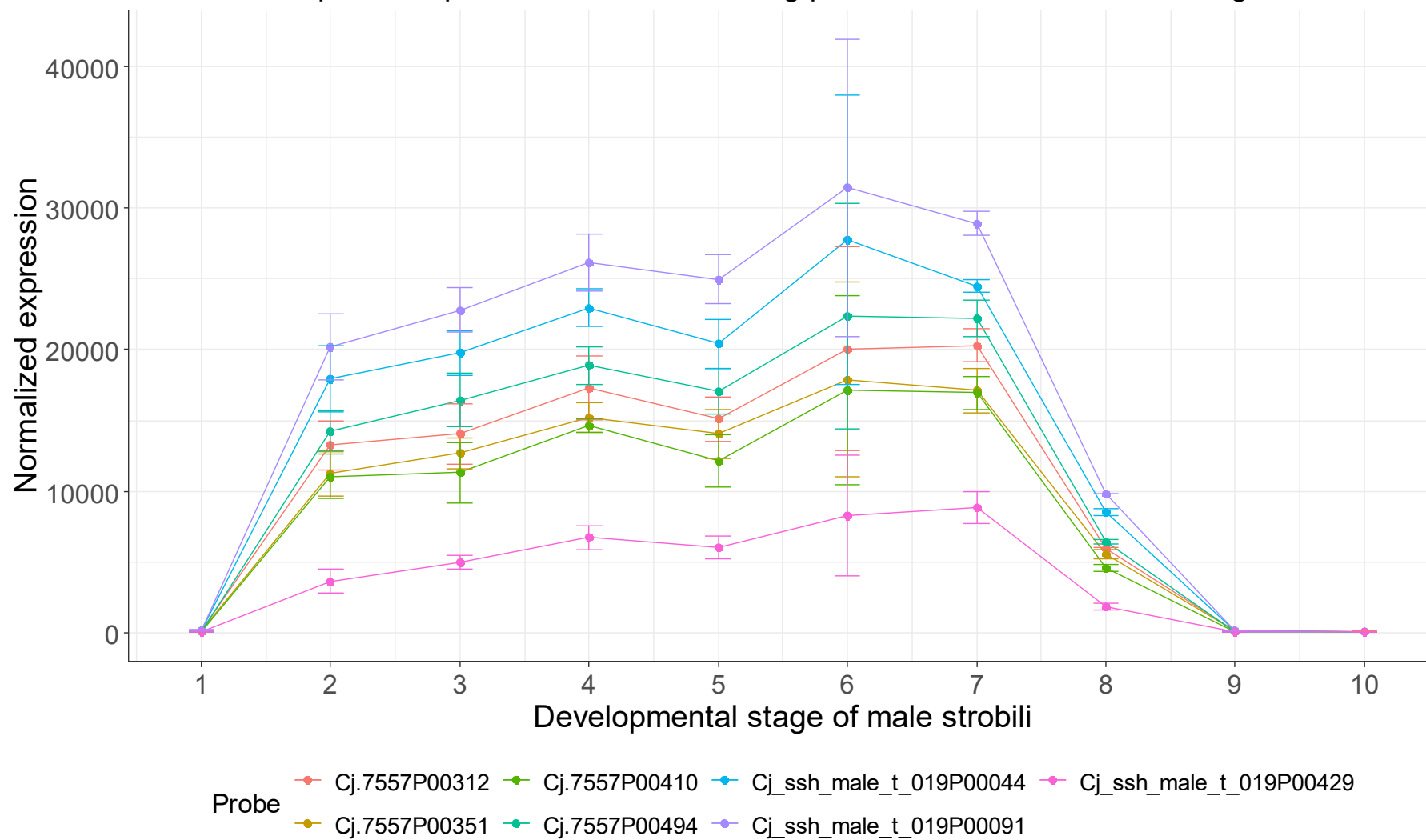

Supplement: Supplementary file 6 — Supplementary Material 6.Supplementary Table 4. CPM values of the GELP candidate gene (SUGI_0493010) across 13 tissues of Cryptomeria japonica. Description of data: Each row corresponds to one RNA-Seq library in SugiExDB. "Sample ID" indicates the SugiExDB library sample identifier, "Tissue" denotes the assigned tissue type, and "CPM" gives the counts-per-million value for the GELP candidate gene (SUGI_0493010). Replicate libraries for the same sample are listed separately. [file 12864_2026_12907_MOESM7_ESM.pdf]

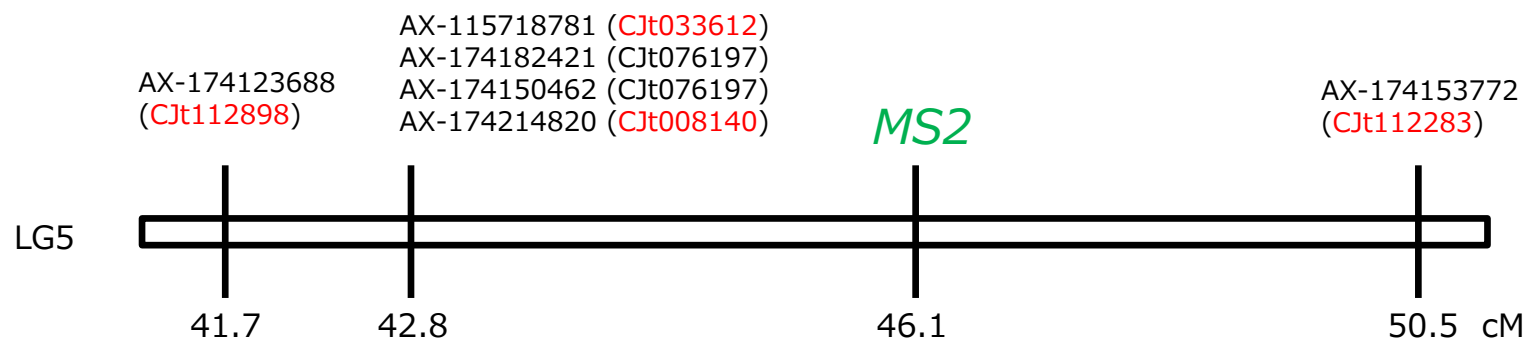

Hasegawa et al.  
(2018)

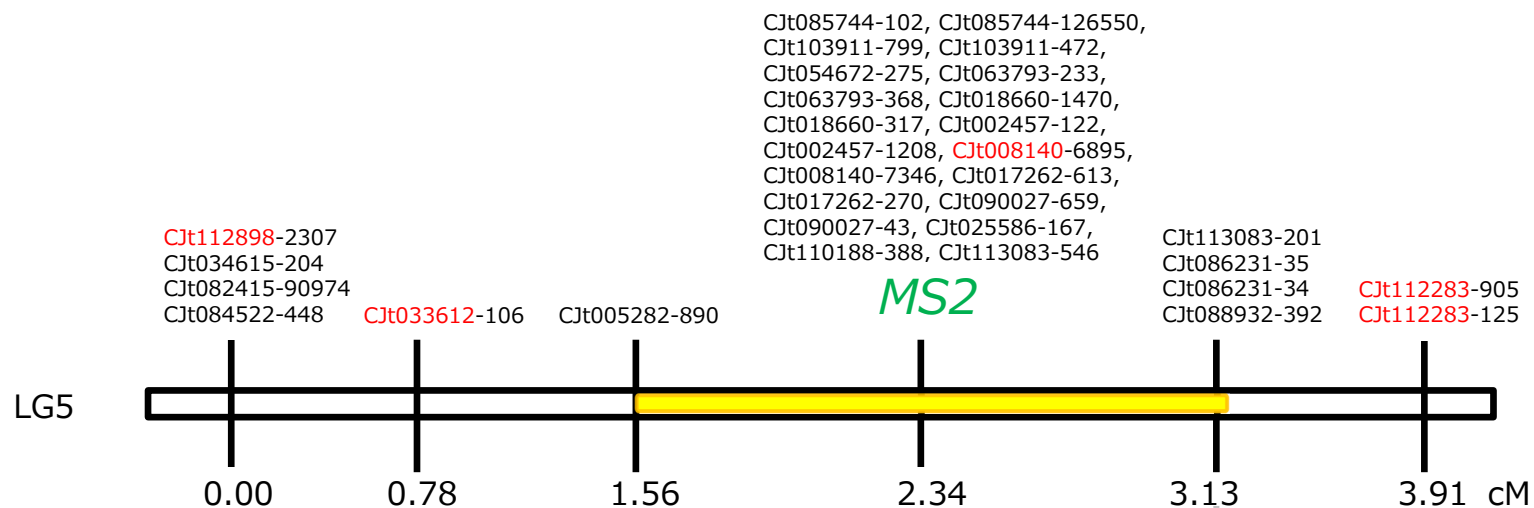

This study  
(2025)

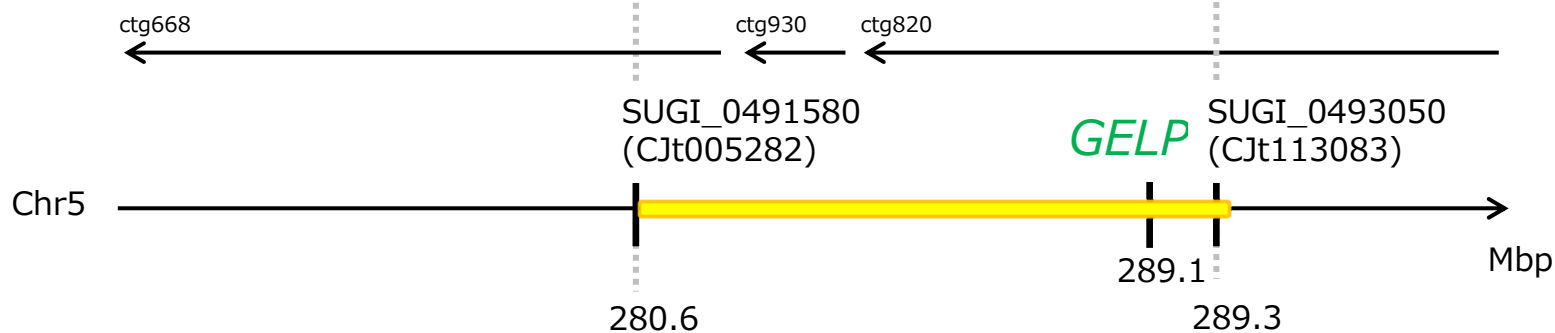

Fujino et al.  
draft ver. 0.1  
(unpublished)

Fujino et al.  
SUGI\_1  
(2024)

Supplement: Supplementary file 7 — Supplementary Material 7.Supplementary Figure 3. Temporal expression profile of SUGI_0493010 in male strobili based on the microarray dataset. Description of data: Normalized signal intensities of microarray probes matching SUGI_0493010 are shown across ten developmental stages of male strobili (mean ± SD). Developmental stages are defined according to Tsubomura et al. (2016) as follows: Stage 1, appearance of scale primordia in the axils; Stage 2, differentiation of microsporangia after scale formation; Stage 3, recognizable microsporangial wall, middle layer, tapetum and pollen mother cells; Stage 4, entry of pollen mother cells into meiosis; Stage 5, completion of meiosis and tetrad formation; Stage 6, degeneration of the callose wall and release of microspores into the microsporangium; Stage 7, degeneration of the tapetum; Stage 8, formation of fibrous bands in the microsporangial wall; Stage 9, pollen mitotic division and maturation of pollen grains; Stage 10, anther dehiscence and pollen release. All probes show a pronounced expression peak at intermediate stages (around Stages 5–7), corresponding to the period of tetrad formation, microspore release, and tapetum degeneration. [file 12864_2026_12907_MOESM4_ESM.pdf]

AT5G41890 expression by tissue (RPKM)

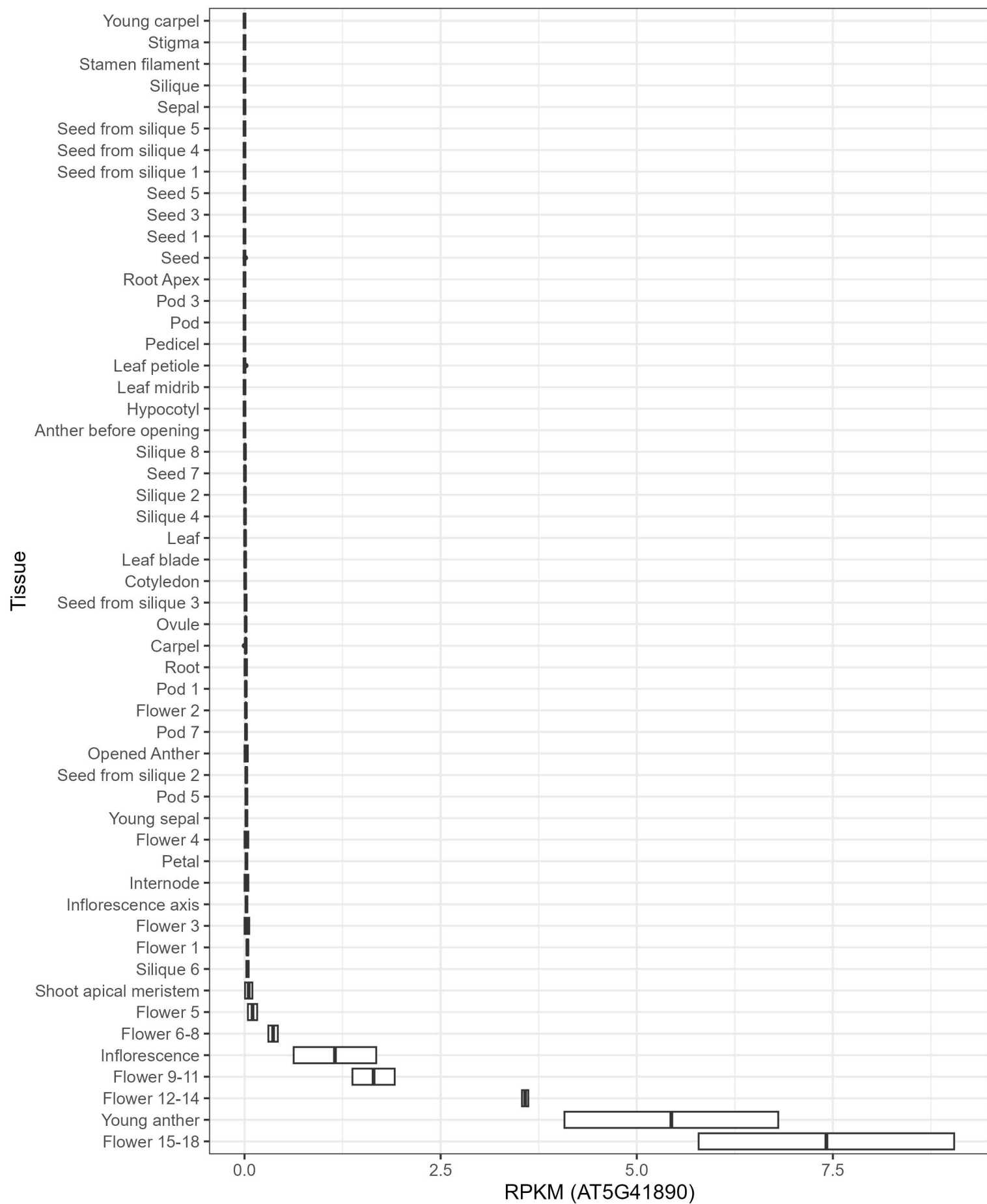

Supplement: Supplementary file 11 — Supplementary Material 11.Supplementary Figure 6. Tissue-specific expression pattern of AT5G41890 in Arabidopsis thaliana based on the RNA-seq atlas. Description of data: Boxplots showing RPKM values of AT5G41890 across multiple tissues in Arabidopsis thaliana, derived from the RNA-seq atlas (Klepikova et al. 2016). For each tissue, all atlas samples annotated with that tissue are summarized; boxes indicate the interquartile range of observed RPKM values. AT5G41890, which represents the closest Arabidopsis GELP homolog to SUGI_0493010 in the phylogenetic analysis, exhibits strongly enriched expression in reproductive tissues, particularly anthers and floral organs, whereas expression is low or undetectable in most vegetative tissues. This reproductive-tissue–biased expression pattern parallels the male-strobilus-specific expression of SUGI_0493010 in Cryptomeria japonica. [file 12864_2026_12907_MOESM11_ESM.pdf]

(A)

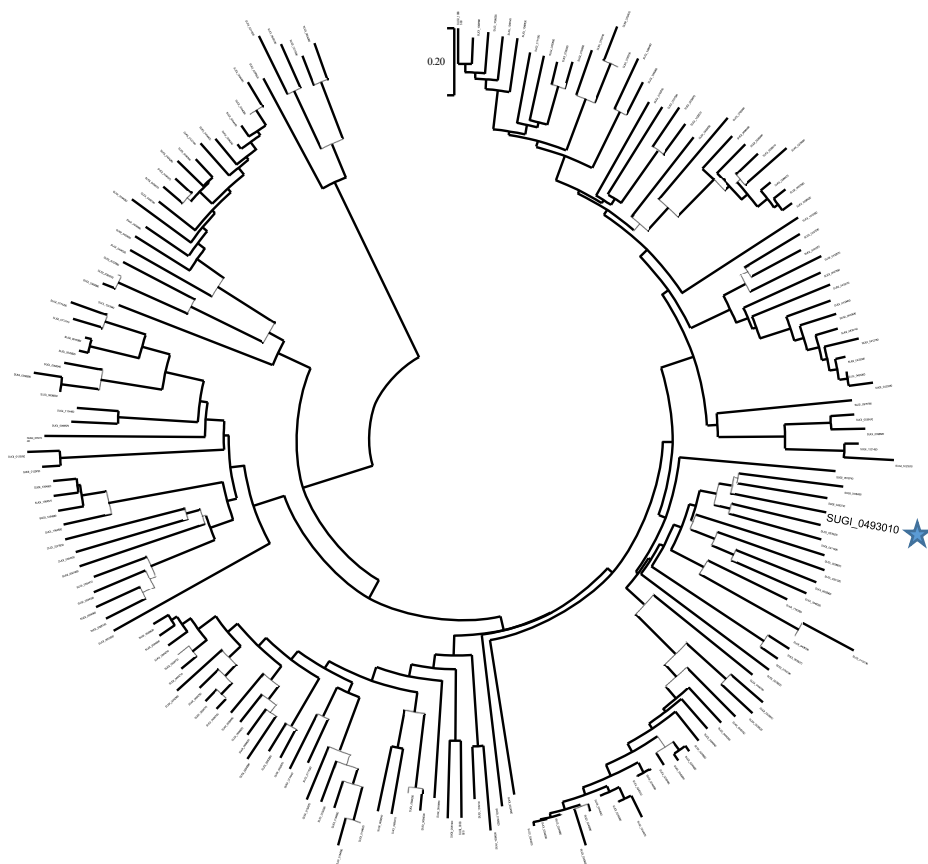

(B)

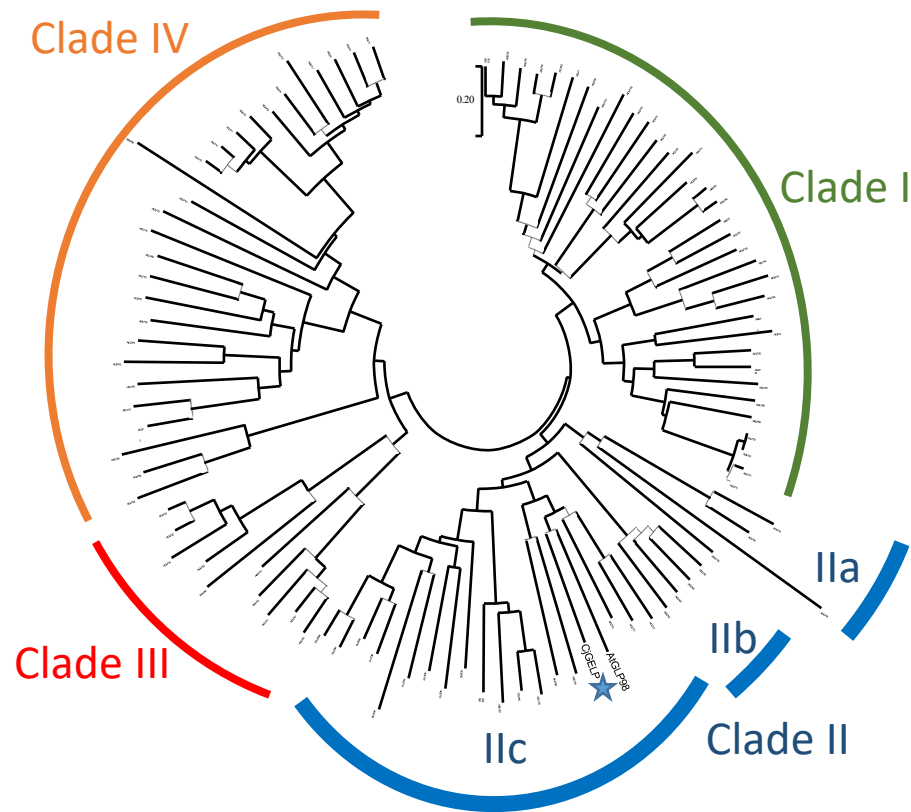

Supplement: Supplementary file 12 — Supplementary Material 12.Supplementary Figure 7. Microscopic cross-section of a male strobilus from a sterile individual (`Y677`) in S1-2 family. Description of data: A partial formation of pollen was observed in the male strobilus of an individual with the ms2/ms2 genotype. The sample was collected from the Chiyoda nursery on 20 December 2023. The scale bar is based on an approximate size of the male strobilus (~4 mm) and is provided as a reference only. [file 12864_2026_12907_MOESM9_ESM.pdf]
