# Supplementary material for: Positional identification of a candidate gene for MALE STERILITY 2 (MS2) by linkage mapping and transcriptomic data in Cryptomeria japonica D. Don
Source: BMC Genomics. 2026 May 19;27:595. doi: 10.1186/s12864-026-12907-4 (PMC13339982; doi:10.1186/s12864-026-12907-4)

(A) *INTS1*

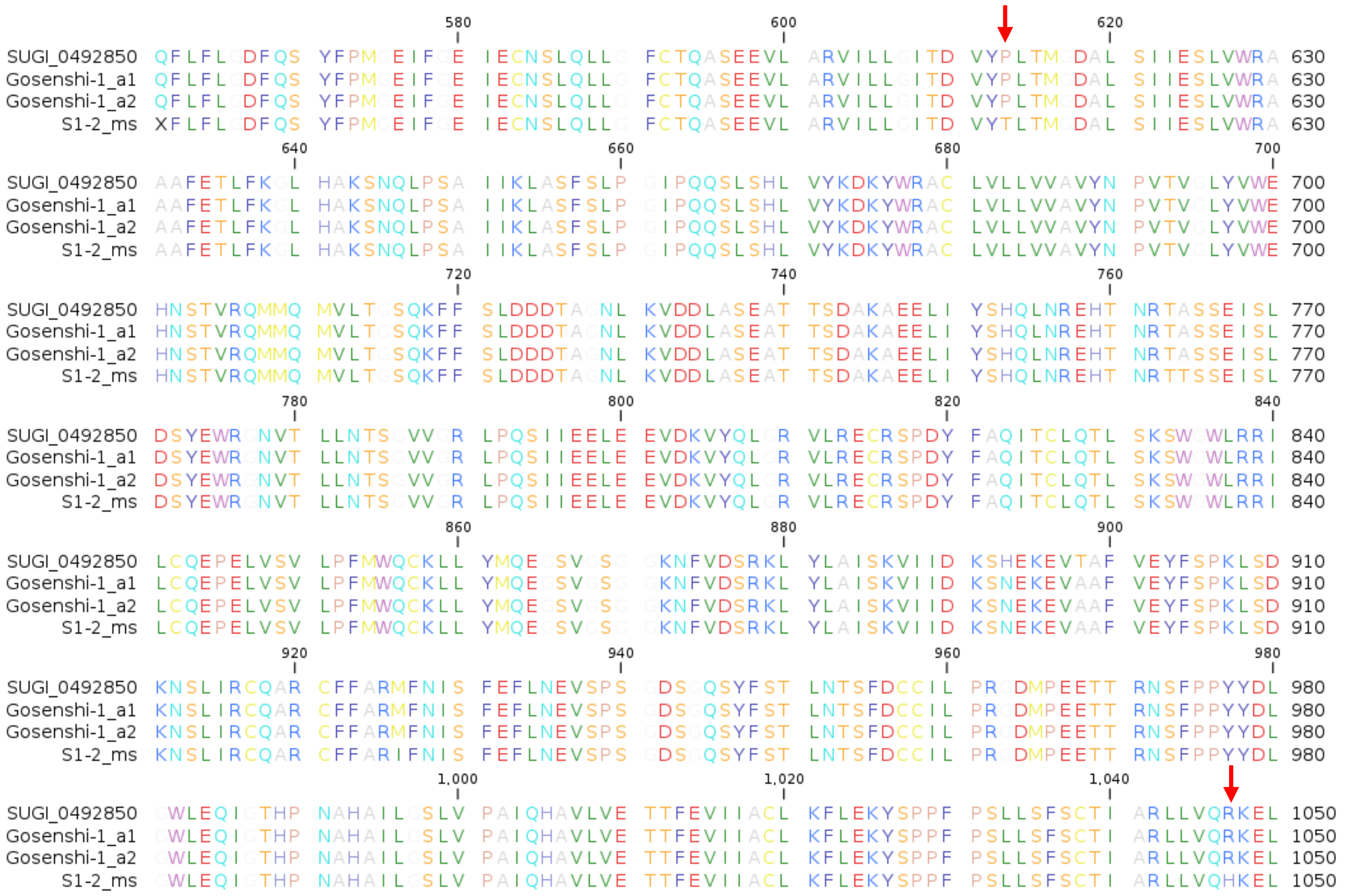

(B) *GELP*

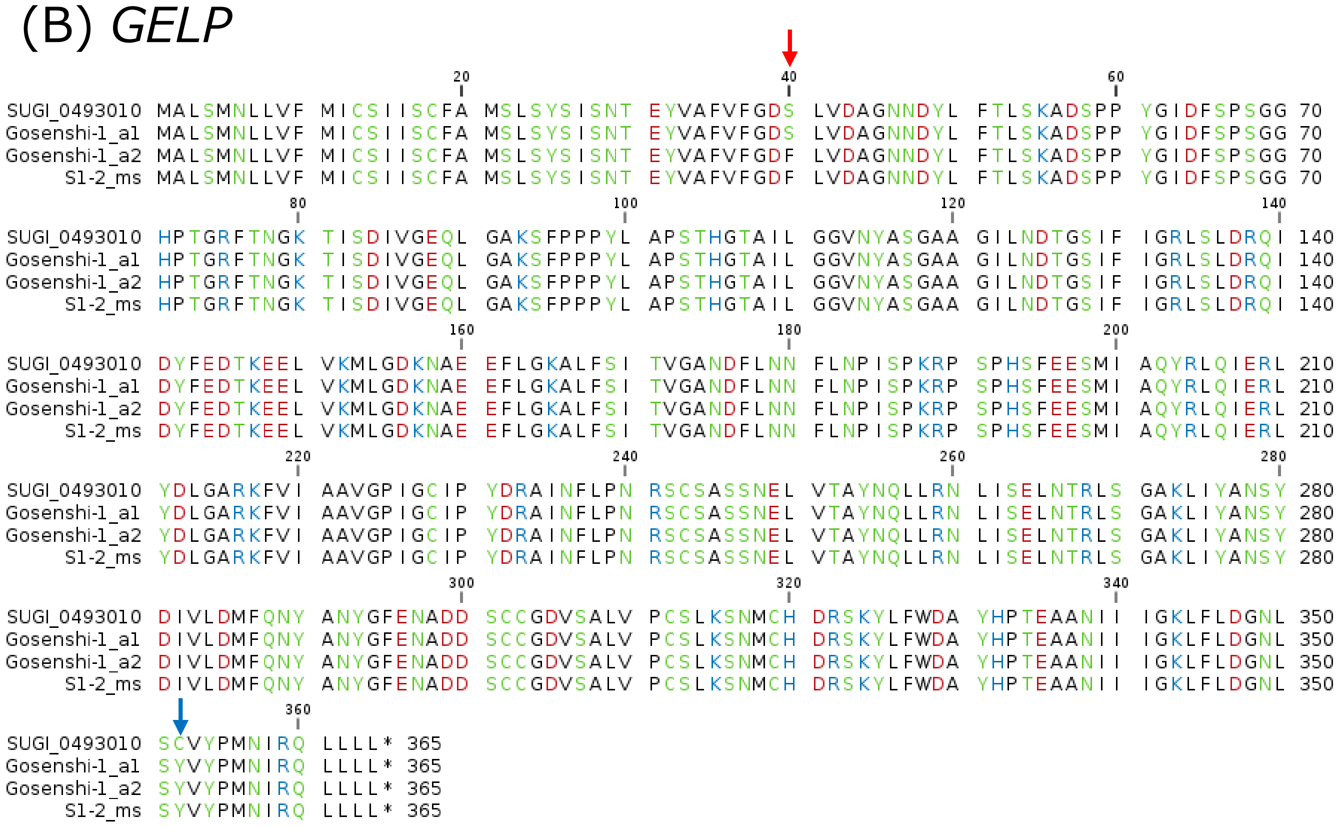

Supplement: Supplementary file 4 — Supplementary Material 4.Supplementary Figure 1. Linkage and physical maps around the MS2 locus. Description of data: Top: Linkage map of LG5 around the MS2 locus reported by Hasegawa et al. (2018), showing Axiom SNP markers and the corresponding CJ3006NRE transcript IDs in parentheses. Middle: Refined linkage map of LG5 obtained in this study. The MS2 region is highlighted in yellow and is delimited by the flanking markers CJt005282-890 and CJt113083-201 with 1.56 cM interval. Bottom: Schematic correspondence between the MS2-linked draft-genome contigs (ctg668, ctg930, and ctg820; Fujino et al., draft genome ver. 0.1, unpublished) and the chromosome-scale reference assembly SUGI_1 (Fujino et al., 2024). The physical MS2 region on chr5 is shown in yellow (280.6–289.3 Mbp), and includes the GELP candidate gene (SUGI_0493010) and its neighboring genes SUGI_0491580 and SUGI_0493050. Genes shown in red are common between Hasegawa et al. (2018) and this study. The yellow interval corresponds to the MS2 region shown in Figure 2. [file 12864_2026_12907_MOESM5_ESM.pdf]
